# Underexplored bacteria as reservoirs of novel antimicrobial lipopeptides

**DOI:** 10.3389/fchem.2022.1025979

**Published:** 2022-10-05

**Authors:** Tanya Clements-Decker, Megan Kode, Sehaam Khan, Wesaal Khan

**Affiliations:** ^1^ Faculty of Health Sciences, University of Johannesburg, Doornfontein, South Africa; ^2^ Department of Microbiology, Faculty of Science, Stellenbosch University, Stellenbosch, South Africa

**Keywords:** lipopeptides, antimicrobial activity, biosynthesis, antibiotics, genome mining, antiSMASH

## Abstract

Natural products derived from microorganisms play a prominent role in drug discovery as potential anti-infective agents. Over the past few decades, lipopeptides produced by particularly *Bacillus*, *Pseudomonas*, *Streptomyces*, *Paenibacillus,* and cyanobacteria species, have been extensively studied for their antimicrobial potential. Subsequently, daptomycin and polymyxin B were approved by the Food and Drug Administration as lipopeptide antibiotics. Recent studies have however, indicated that *Serratia*, *Brevibacillus,* and *Burkholderia*, as well as predatory bacteria such as *Myxococcus*, *Lysobacter,* and *Cystobacter*, hold promise as relatively underexplored sources of novel classes of lipopeptides. This review will thus highlight the structures and the newly discovered scaffolds of lipopeptide families produced by these bacterial genera, with potential antimicrobial activities. Additionally, insight into the mode of action and biosynthesis of these lipopeptides will be provided and the application of a genome mining approach, to ascertain the biosynthetic gene cluster potential of these bacterial genera (genomes available on the National Center for Biotechnology Information) for their future pharmaceutical exploitation, will be discussed.

## Introduction

Microbial natural products are valuable sources of structurally diverse, antimicrobial compounds; many of which are currently implemented as therapeutic agents (Genilloud, 2014; Challinor and Bode, 2015). However, microbial resistance to several classes of commercial antimicrobials has compromised the successful treatment of infectious diseases, resulting in significant morbidity and mortality rates worldwide (Genilloud, 2014; Murray et al., 2022). There is thus an urgent need to not only discover new lead antimicrobial classes that effectively combat multidrug-resistant pathogens, but to integrate innovative approaches and technologies into antimicrobial drug discovery (Genilloud, 2014). A prominent class of microbial natural products that has gained interest over the last several decades, due to their potent bioactive properties, is lipopeptides (Patel et al., 2015).

Structurally, lipopeptide antibiotics are characterised by a core hydrophilic head-group of amino acids considered as the peptide moiety, linked to a hydrophobic fatty acid (FA) chain, resulting in their amphiphilic nature (Cochrane and Vederas, 2016; Reynolds et al., 2018). A wide variety of lipid chains has been observed, including variation in their chain length, configuration, and degree of unsaturation, while variation in the peptide moiety is due to the change in amino acid composition, which may be cyclic or linear (Burgard et al., 2017; Reynolds et al., 2018). This variation in the structural diversity of lipopeptides results in a broad range of beneficial biological properties, including antibacterial, antiviral, antifungal, antitumor and immunomodulator activities (Patel et al., 2015).

The isolation and characterisation of amphomycin (produced by a *Streptomyces canus* strain) over 60 years ago, then pioneered the usage of lipopeptides as antibiotics (Heinemann et al., 1953; Reynolds et al., 2018). Subsequently, the class of lipopeptides expanded, where prospecting for new lipopeptides primarily focused on exploiting a relatively small group of bacterial genera, including *Streptomyces*, *Bacillus*, *Pseudomonas*, *Paenibacillus,* and cyanobacteria (Patel et al., 2015; Li et al., 2021a). In 2003, the cyclic lipopeptide antibiotic, daptomycin (also called Cubicin^®^), produced by *Streptomyces roseosporus,* was approved by the Food and Drug Administration for the treatment of serious skin and soft tissue infections, and in 2006, for the treatment of methicillin-resistant *Staphylococcus aureus* (*S*. *aureus*; MRSA) bacteraemia (Strieker and Marahiel, 2009; Cochrane and Vederas, 2016). Moreover, several different lipopeptides, such as tsushimycin, daptomycin and surfactin, amongst others, have also shown promise as antiviral drug candidates against the coronavirus, SARS-CoV-2 (Chowdhury et al., 2021; De Vries et al., 2021).

Recently, several bacterial genera such as *Serratia*, *Brevibacillus* and *Burkholderia*, have emerged as underexplored sources of novel antimicrobial lipopeptides (Etzbach et al., 2014; Burgard et al., 2017; Ganley et al., 2018; Li et al., 2018; Sang et al., 2019; Ning et al., 2021). For example, four species within the *Serratia* genus, including *Serratia marcescens* (*S. marcescens*)*, Serratia plymuthica* (*S. plymuthica*)*, Serratia ureilytica* (*S. ureilytica*), and *Serratia surfactantfaciens* (*S. surfactantfaciens*), have been reported as lipopeptide producers, with serrawettin W1 (also referred to as serratamolide), serrawettin W2 or stephensiolides families, produced by various strains (Kuo et al., 2012; Menezes et al., 2021; Clements-Decker et al., 2022). Strains of *Brevibacillus laterosporus* (*B. laterosporus*) and *Brevibacillus brevis* (*Br. brevis*) also produce several lipopeptide families, such as tauramamides, bogorols, brevibacillins, brevilaterins, brevicidines, laterocidines, relacidines, brevistin and surfactin (Barsby et al., 2006; Desjardine et al., 2007; Wang et al., 2010; Yang et al., 2016; Li et al., 2018, 2020a, 2020b; Ning et al., 2021). Moreover, *Burkholderia* species have emerged as promising lipopeptide producers, as several species within the genus [including *Burkholderia ambifaria* (*B. ambifaria*)*, Burkholderia pseudomallei* (*B. pseudomallei*)*, Burkholderia plantarii* (*B. plantarii*)*, Burkholderia gladioli* (*B. gladioli*)*,* and *Burkholderia glumae* (*B. glumae*)] produce burkholdines, icosalides, haereogladins, haereoglumins, haereogladiodins, haereoplantins, burriogladins, burrioglumins, burriogladiodins, burrioplantins, malleipeptins and glidopeptins families (Tawfik et al., 2010; Biggins et al., 2014; Dose et al., 2018; Niehs et al., 2018; Thongkongkaew et al., 2018; Wang et al., 2018; Yoshimura et al., 2020; Chen et al., 2021).

Predatory bacteria have also been recognised as natural factories of bioactive compounds, as their lifestyle depends on the production of various enzymes and secondary metabolites to naturally invade and consume specific prey microorganisms as a nutrient source (Rosenberg and Varon, 1984; Herencias et al., 2020; Sester et al., 2020; Atterbury and Tyson, 2021). Certain predatory bacterial genera, such as such as *Myxococcus*, *Cystobacter,* and *Lysobacter*, have then been identified to produce lipopeptides during secondary metabolism (Herrmann et al., 2017; Yan et al., 2018; Yue et al., 2022). For example, one of the most extensively studied lipopeptides produced by *Myxococcus* species [including *Myxococcus virescens* (*M. virescens*)*, Myxococcus xanthus* (*M. xanthus*) and an unclassified *Myxococcus* sp.], are the myxochromides, whilst cystomanamides from *Cystobacter* species [including *Cystobacter fuscus* (*C. fuscus*)], have also been structurally elucidated (Etzbach et al., 2014). In addition, *Lysobacter* have recently been highlighted as a promising source of lipopeptides, as various species within the genus [including *Lysobacter enzymogenes* (*L. enzymogenes*), *Lysobacter antibioticus* (*L. antibioticus*)*, Lysobacter capsica* (*L. capsica*) and an unclassified *Lysobacter* sp.] were reported as lipopeptide producers, with WAP-8294A, WBP-29479A1, lysocin and tripropeptin families described (Sang et al., 2019; Arlt et al., 2021).

Genetically, these lipopeptide classes are primarily synthesised non-ribosomally during secondary metabolism by complex enzymes known as non-ribosomal peptide synthetases (NRPSs) and polyketide synthases (PKSs) that are encoded by large biosynthetic gene clusters (BGCs) (Aleti et al., 2015). Although a recent report has identified ribosomally synthesised lipopeptides (Hubrich et al., 2022), this review will primarily focus on lipopeptides synthesised non-ribosomally. The development of high-throughput whole genome sequencing technology has made it possible to identify new BGCs within genomic data and thus putatively predict structures of their associated chemical products (Kreutzer and Nett, 2012; Aleti et al., 2015). This circumvents the need to rely on bioactive-guided screening assays for the discovery of novel compounds and has subsequently promoted a paradigm shift in natural product research (Kreutzer and Nett, 2012; Aleti et al., 2015). Moreover, advances in next generation sequencing have contributed to the surge of genome sequences available in public databases. Therefore, genome mining in combination with available web-based tools used for metabolite prediction, such as antibiotics and Secondary Metabolite Analysis Shell (antiSMASH), can be employed to reveal uncharacterised secondary metabolites, including lipopeptides, within the genomes of relatively unexploited bacterial genera (Aleti et al., 2015).

This review thus highlights the antimicrobial lipopeptides produced by members of the *Serratia*, *Brevibacillus*, *Burkholderia*, *Myxococcus*, *Cystobacter,* and *Lysobacter* genera that have been described to date and their subsequent antimicrobial activity and mode of action. In addition, a global genome mining approach, based on the antiSMASH web tool, was applied for the exploration of lipopeptide BGCs within the genomes of the six selected bacterial genera as available on the National Center for Biotechnology Information (NCBI) database, highlighting the various species as underexplored sources for new lipopeptide families.

## Antimicrobial lipopeptides: Production by underexplored bacterial genera

### Serratia

**FIGURE 1:**
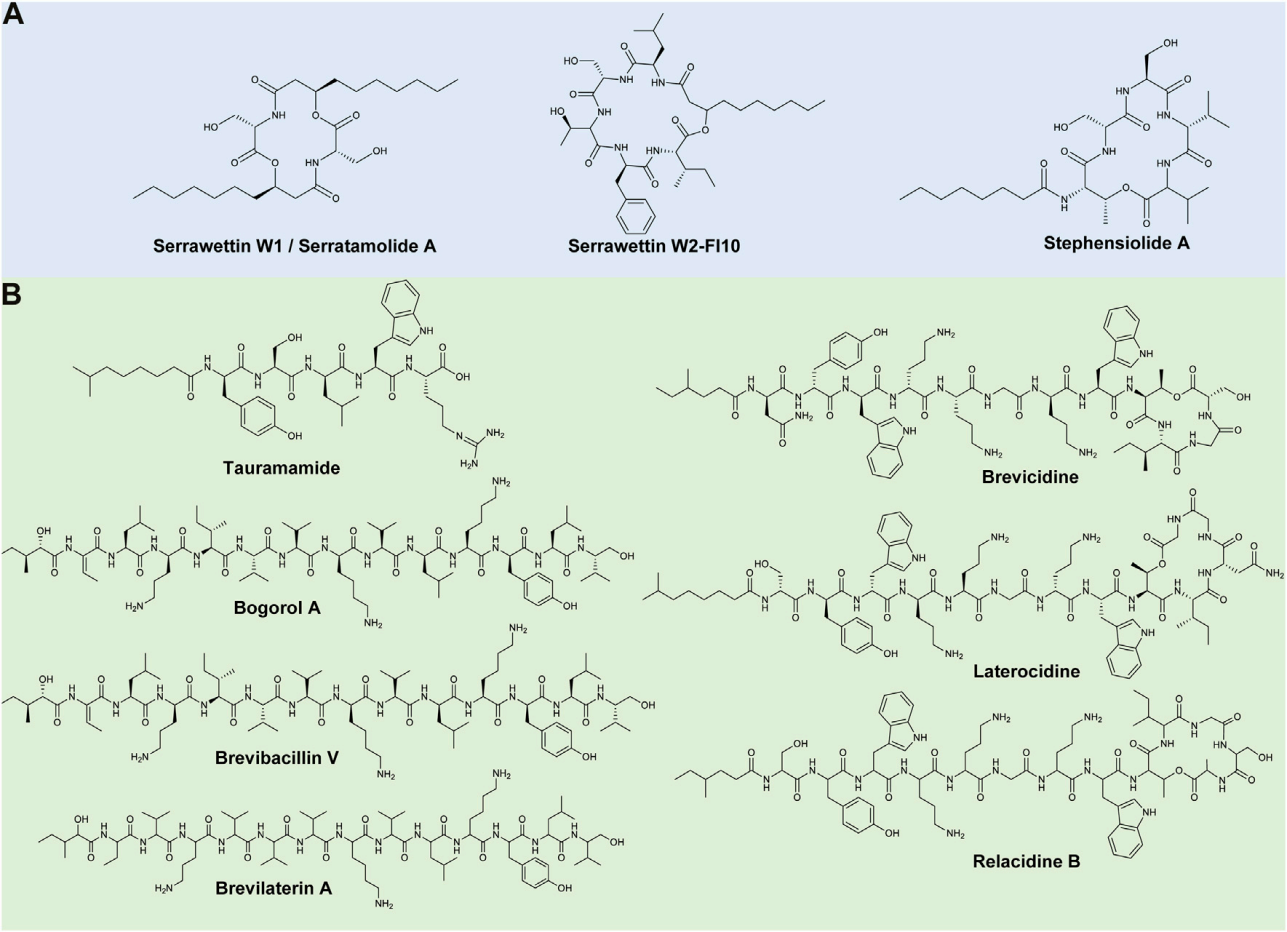
Representative lipopeptides produced by **(A)** *Serratia* and **(B)** *Brevibacillus* species. The structural information was obtained from the PubChem database (https://pubchem.ncbi.nlm.nih.gov/), as well as the corresponding references provided in-text. The structures were subsequently drawn in ChemDraw Ultra version 12.0.

Members of the *Serratia* genus are Gram-negative, facultative anaerobes and belong to the Enterobacteriaceae family (Grimont and Grimont, 2015). The genus is comprised of 23 validly published species (available at: https://lpsn.dsmz.de/genus/serratia) that occur ubiquitously in water, soil, plants, insects, and marine environments (Cristina et al., 2019). Although originally considered non-pathogenic saprophytic bacteria, certain species within this genus, such as *S. marcescens, Serratia fonticola* (*S. fonticola*), and *S. plymuthica,* have been implicated as important nosocomial pathogens (Carrero et al., 1995; Cristina et al., 2019; Hai et al., 2020). *Serratia* species do, however, have a notable secondary metabolism, as they can produce a wide range of antimicrobial natural products, such as the well-known red pigment called prodigiosin, whilst also showing promise as lipopeptide producers (Clements et al., 2019). To date, three lipopeptide families, as well as numerous analogues of these lipopeptides families, have been discovered, including serrawettin W1 (also referred to as serratamolide), serrawettin W2, and stephensiolides (Figure 1A).

Serratamolide was first discovered by Wasserman et al. (1961), after it was produced by a *Serratia* strain, and was later isolated from a *S. marcescens* strain where the lipopeptide was referred to as serrawettin W1 (Matsuyama et al., 1985) (Figure 1A). This lipopeptide has a peptide moiety of two amino acid residues (i.e., Ser-Ser) linked to two C_10_ fatty acid chains (Figure 1A) (Matsuyama et al., 1985). Numerous analogues of this compound have been identified, which are produced by pigmented and non-pigmented *S. marcescens, S. ureilytica,* and *S. plymuthica* strains (as well as a *Serratia liquefaciens* strain that was reclassified as a *S. marcescens* strain), that vary based on the length and saturation/unsaturation of the fatty acid moiety and can be cyclic or open-ring structures (Eberl et al., 1999; Labbate et al., 2007; Dwivedi et al., 2008; Kuo et al., 2012; Clements et al., 2021; Menezes et al., 2021). Serratamolides exhibit promising activity against Gram-positive bacteria, such as *Enterococcus faecalis*, *Mycobacterium* spp. and MRSA (Dwivedi et al., 2008; Kadouri and Shanks 2013; Clements et al., 2021).

Serrawettin W2 is a cyclic lipopeptide family produced by *S. marcescens* and *S. surfactantfaciens*. The peptide moiety is comprised of five amino acids (i.e., Leu-Ser-Thr-Phe-Ile) attached to a C_10_ fatty acid chain, now referred to as serrawettin W2-FI10 (Figure 1A) (Matsuyama et al., 1986; Clements-Decker et al., 2022). Analogues of serrawettin W2 (i.e., sw-1–sw-4 and sw-6–sw-8) were putatively identified and differed based on amino acid substitutions (first, second or fifth amino acid positions) (Su et al., 2016). Similarly, Motley et al. (2017) elucidated the structures of three new analogues (i.e., W4–W6) with varying fatty acyl chain lengths and amino acid compositions (fourth or fifth amino acid positions). The serrawettin W2 family was recently expanded, and the nomenclature clarified, as 16 new analogues (such as W2-YV8, W2-Fl8, W2-FV10, W2-FL12:1, etc.) were isolated from a *S. marcescens* strain (Clements-Decker et al., 2022), which differed based on position of the final two amino acids and varying fatty acid chain length and saturation/unsaturation (Clements-Decker et al., 2022). Serrawettin W2 analogues have also exhibited promising activity against Gram-positive bacteria, such as MRSA [minimum inhibitory concentration (MIC) of 4 μg/ml] and *Enterococcus faecium* (*E. faecium*) (MIC of 15.6 μg/ml) and Gram-negative bacteria, such as *Pseudomonas aeruginosa* (*P. aeruginosa*) (Su et al., 2016; Heise et al., 2019; Clements-Decker et al., 2022). Serrawettin W3 was originally partially characterised as its own lipopeptide family and contained a fatty acid chain (dodecanoic acid) and five amino acids, including Thr, Ser, Val, Leu and Ile (Matsuyama et al., 1986). However, Clements-Decker et al. (2022) has since suggested that W3 is an analogue of serrawettin W2-FI10 based on metabolomics and genome mining analysis.

Stephensiolides are a cyclic lipopeptide family produced by various *Serratia* sp. (Ganley et al., 2018). Stephensiolide A is comprised of a peptide moiety of five amino acids of Thr-Ser-Ser-Val-Val attached to a C_8_ fatty acid chain (Matsuyama et al., 1986) (Figure 1A). Analogues (stephensiolides B–K) of this compound were identified, varying in the final two amino acids residues and length and saturation/unsaturation of the fatty acid chain (Ganley et al., 2018). Moreover, these lipopeptides exhibit promising activity against Gram-positive bacteria, such as *Bacillus subtilis* (*B. subtilis*) [half-maximal inhibitory concentration (IC_50_) of 15 μg/ml], as well as antiparasitic activity against *Plasmodium falciparum* (IC_50_ of 14 μg/ml) (Ganley et al., 2018).

### Brevibacillus

Members of the *Brevibacillus* genus are rod-shaped, Gram-positive or Gram-variable firmicutes that belong to the Paenibacillaceae family (De Vos et al., 2011; Panda et al., 2014). The endospore-forming *Brevibacillus* species were genetically reclassified as a distinct genus from the *Bacillus brevis (B. brevis)* cluster in 1996 (Ray et al., 2020), and now consists of 29 validly published species (available at: https://lpsn.dsmz.de/genus/brevibacillus). Members of this genus are widely distributed in nature, occurring in soil, plants, aquatic environments, intestinal tracts of insects, and animals (Panda et al., 2014; Yang and Yousef, 2018). Although certain *Brevibacillus* species can cause infections in immunocompromised individuals, such as *Br. brevis* and *B. laterosporus*, they are rarely implicated as human pathogens (Parvez et al., 2009; Curtis et al., 2020). *Brevibacillus* species are also well-known producers of antimicrobial peptides, such as gramicidins and tyrocidines (Yang and Yousef, 2018), while members of this genus have shown promise as lipopeptide producers. Eight lipopeptide families have been identified, including tauramamides, bogorols, succilins, brevibacillins, brevilaterins, brevicidines, laterocidines and relacidines. A *Br. brevis* strain was then found to produce surfactin, a lipopeptide commonly isolated from *Bacillus* species (Wang et al., 2010). Additionally, Singh et al. (2021) identified an iturin-like lipopeptide with a structure of FA-Asp-Asp-His-Ser-Ala-Gly-Thr from *Brevibacillus* sp. GI9. Moreover, a *Br. brevis* strain (previously *B. brevis*) was found to produce an acylpeptide, namely brevistin, with a structure of FA-Thr-Dab-Asp-Gly-Asn-Asp-Gly-Trp-Ile-Dab-Phe (where Dab refers to diaminobutanoic acid) in 1975, while no recent information on this antimicrobial lipopeptide have been reported and it will thus not be discussed in this review (Shoji et al., 1976).

Tauramamide is a linear lipopeptide produced by a marine *B. laterosporus* strain (Desjardine et al., 2007) and is comprised of five amino acid residues (i.e., Tyr-Ser-Leu-Trp-Arg) linked to a C_6_ fatty acid chain (Figure 1B). Homologues of tauramamide were also elucidated and resulted from the addition of methyl or ethyl esters (Desjardine et al., 2007). Tauramamide and tauramamide ethyl ester exhibit potent activity (MIC of 0.1 μg/ml) against *Enterococcus* sp., while tauramamide ethyl ester has also been reported to display weak activity against *Candida albicans* (*C. albicans*) (Desjardine et al., 2007).

Bogorols, brevibacillins and brevilaterins are structurally similar, linear lipopeptide families produced by *B. laterosporus* strains (Barsby et al., 2001, 2006; Yang et al., 2016; Ning et al., 2021). All three lipopeptide families consist of 13 amino acids linked to a C_6_-fatty acid chain, which differ based on the peptide moiety. Bogorol A, brevibacillin and brevilaterin A have peptide sequences of Dhb-Leu-Orn-Ile-Val-Val-Lys-Val-Leu-Lys-Tyr-Leu-valinol, Dhb-Leu-Orn-Ile-Ile-Val-Lys-Val-Val-Lys-Tyr-Leu-valinol, and Aba-Val-Orn-Val-Val-Val-Lys-Val-Leu-Lys-Tyr-Leu-valinol, respectively (Figure 1B) (Barsby et al., 2001, 2006; Yang et al., 2016; Ning et al., 2021). Analogues of bogorol A have been identified (bogorol B–E, B-JX, I–L), differing based on amino acid substitutions (amino acid positions of 2–5 and 9) (Barsby et al., 2006; Jiang et al., 2017; Li et al., 2020a). Succinylated bogorols (addition of a succinyl group to the third amino acid residue in bogorols I–L), named succilins, have additionally been identified (Li et al., 2020a). Moreover, Singh et al. (2021) identified a bogorol-like lipopeptide with a structure of Dhb-Tyr-Orn-Ile-Val-Val-Lys-Val-Leu-Asp-Val-Glu from *Brevibacillus* sp. SKDU10. In comparison, three analogues of brevibacillin have been identified, namely brevibacillin V, 2V, and I, and differ based on amino acid substitutions (amino acid positions of 4, 5, and 8) (Wu et al., 2019; Zhao et al., 2021). Finally, analogues of brevilaterin A (i.e., B–E, X, V1–V6) have been identified and differ based on amino acid substitutions (amino acid positions of 2–4, and 6) (Ning et al., 2021; Chen et al., 2022). All three families display potent activity against Gram-positive bacteria, such as *S. aureus* strains (MICs of 1–5 μg/ml) (Barsby et al., 2006; Ning et al., 2021; Zhao et al., 2021) as well as moderate activity against Gram-negative bacteria, such as *Escherichia coli* (*E. coli*) (MICs of 16–75 μg/ml) (Barsby et al., 2006; Zhao et al., 2021; Chen et al., 2022). The antifungal activity of these lipopeptide families has additionally been reported (Yang et al., 2016; Jiang et al., 2017; Wu et al., 2019, 2021; Ning et al., 2021; Chen et al., 2022).

Brevicidines are partially cyclised lipopeptides produced by *B. laterosporus* strains (Li et al., 2018). Brevicidine is comprised of 12 amino acids (i.e., Asn-Tyr-Trp-Orn-Orn-Gly-Orn-Trp-Thr-Ile-Gly-Ser), with the final four amino acids cyclised via a lactone bond and linked to a fatty acyl chain (i.e., 4-methyl-hexanoyl) (Figure 1B) (Li et al., 2018; Hermant et al., 2021). An analogue, namely brevicidine B, was then identified by Zhao and Kuipers (2021) and contained a single amino acid substitution (Tyr^2^ to Phe^2^). The brevicidines exhibit potent activity against Gram-negative pathogens, such as *E. coli* and *Klebsiella pneumoniae* (MICs of 0.25–4 μg/ml), amongst others (Zhao and Kuipers, 2021). Brevicidine also exhibits moderate activity against *B. subtilis* (MIC of 32 μg/ml), while brevicidine B displayed potent activity against vancomycin-resistant *Enterococcus* (VRE) and MRSA strains (MICs of 2–8 μg/ml) (Li et al., 2018; Zhao and Kuipers, 2021).

Laterocidine and relacidines (A and B) are partially cyclised lipopeptides produced by *B. laterosporus* strains (Li et al., 2018; 2020b). Both lipopeptide families have 13 amino acids with the final five amino acids cyclised via a lactone bond, linked to a fatty acyl chain (i.e., 7-methyl-octanoyl or 4-methyl-hexanoyl) (Li et al., 2018; Hermant et al., 2021). These lipopeptides differ based on the peptide moiety, where laterocidine has a peptide sequence of Ser-Tyr-Trp-Orn-Orn-Gly-Orn-Trp-Thr-Ile-Asn-Gly-Gly (Figure 1B), while relacidine A has a peptide sequence of Ser-Tyr-Trp-Orn-Orn-Gly-Orn-Trp-Thr-Ile-Gly-Ser-Gly. Relacidine B varies from A based on a single amino acid substitution of Gly^13^ to Ala^13^ (Figure 1B). Laterocidine and relacidines have exhibited promising activity against Gram-negative bacteria, such as *Xanthomonas campestris* (*X. campestris*)*, E. coli* and *P. aeruginosa*, with MICs of 0.25–8 μg/ml recorded (excluding laterocidine against *X. campestris,* where the MIC of was not tested) (Li et al., 2018; 2020b). However, moderate or no activity was observed against Gram-positive bacteria (Li et al., 2018; 2020b).

### Burkholderia

The Gram-negative *Burkholderia* genus is comprised of 34 validly published species (available at: https://lpsn.dsmz.de/genus/burkholderia) and belongs to the Burkholderiaceae family (Garrity et al., 2005). *Burkholderia* species are widely distributed in aquatic and soil niches, while also associating with eukaryotic hosts such as plants, fungi, animals, and humans (Coenye and Vandamme, 2003; Kunakom and Eustáquio, 2019). In particular, members of the *Burkholderia cepacia* (*B. cepacia*) complex, *Burkholderia thailandensis* (*B. thailandensis*), *Burkholderia pickettii*, *B. gladioli*, *B. pseudomallei,* and *Burkholderia mallei* (*B. mallei*) have been implicated as opportunistic human pathogens (Fernández et al., 1996; Graves et al., 1997; Srinivasan et al., 2001; Chang et al., 2017; Kunakom and Eustáquio, 2019). *Burkholderia* species have relatively large genome sizes, split up into three chromosomes and large plasmids, which contain BGCs encoding for diverse secondary metabolites, such as the lipopeptide families (Esmaeel et al., 2016). The lipopeptide families that have been discovered and characterised from *Burkholderia* species include burkholdines, icosalides, haereogladins, haereoglumins, haereogladiodins, haereoplantins, burriogladins, burrioglumins, burriogladiodins, burrioplantins, malleipeptins, and glidopeptins. Lipopeptides from *Paraburkholderia* species, such as holrhizin and rhizomide, as well as lipoglycopeptides (such as cepacidine from *B. cepacia*) will however, not be discussed in this review (Niehs et al., 2018; Wang et al., 2018).

**FIGURE 2:**
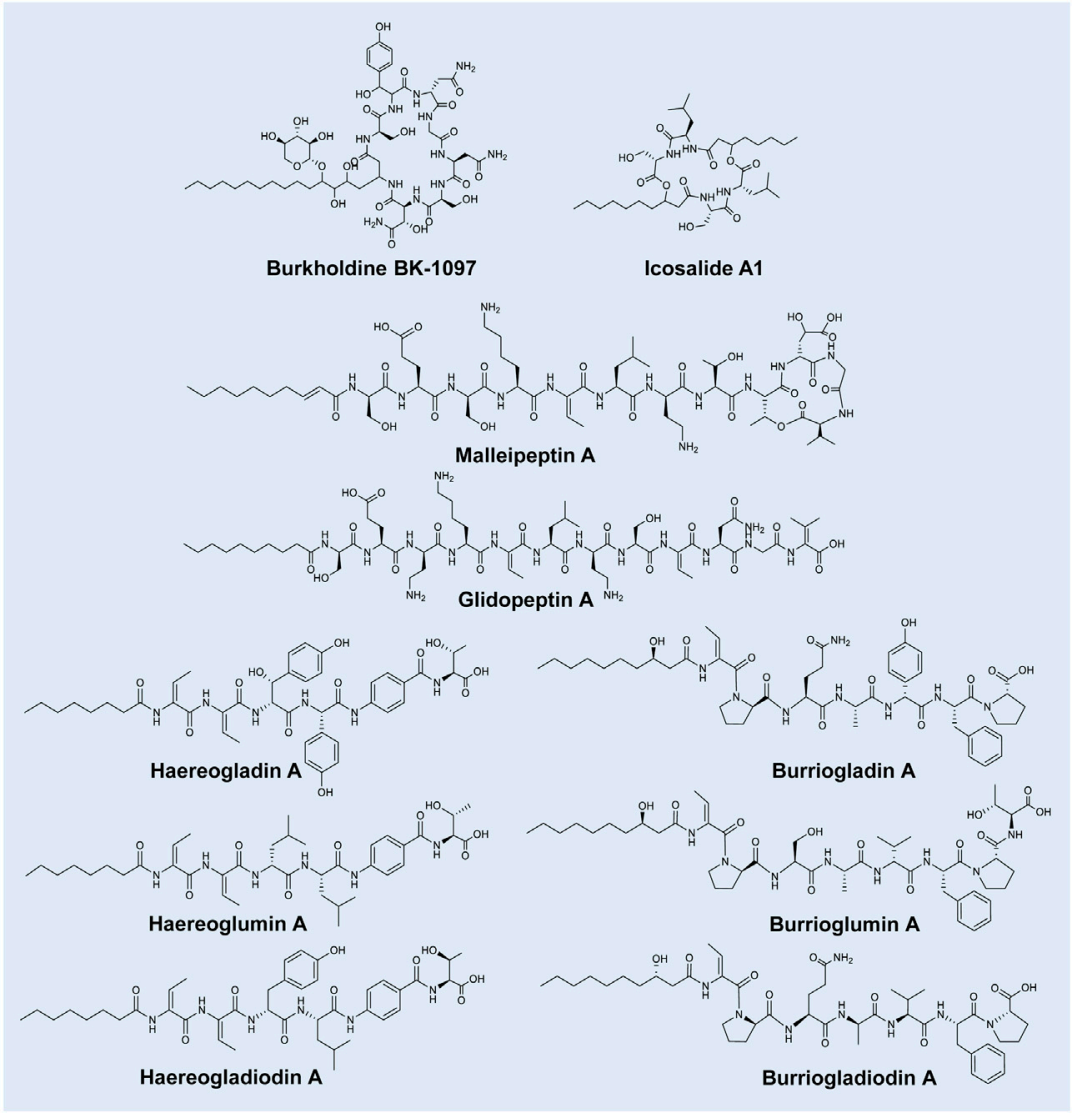
Representative lipopeptides produced by *Burkholderia* species. The structural information was obtained from the PubChem database (https://pubchem.ncbi.nlm.nih.gov/), as well as the corresponding references provided in-text. The structures were subsequently drawn in ChemDraw Ultra version 12.0.

Burkholdines are cyclic lipopeptides primarily produced by *B. ambifaria* and are comprised of a peptide moiety of Asn-Ser-Asn-Gly-Asn-Tyr-Ser attached to a fatty acid chain (referred to as Bk-1097; Figure 2) (Lu et al., 2009; Tawfik et al., 2010; Ellis et al., 2012). Analogues of Bk-1097 have since been identified (i.e., Bk-1229, Bk-1119, Bk-1213 and Bk-1215) and differ based on an additional xylose attached to the fatty acid chain (a cyclic glyco-lipopeptide) or amino acid substitutions (Lu et al., 2009; Ellis et al., 2012; Lin et al., 2012). The five burkholdines have been shown to exhibit promising antifungal properties, particularly against *Saccharomyces cerevisiae*, *C. albicans* and *Aspergillus niger* (MICs of 0.1–31 μg/ml) (Tawfik et al., 2010; Lin et al., 2012).

Icosalides are a family of cyclic two-tailed depsipeptides reportedly produced by a fungal *Aureobsidium* sp. (Boros et al., 2006). Dose et al. (2018) then isolated a *B. gladioli* strain capable of producing these lipopeptides. Thus, the origin of icosalides was deliberated by Dose et al. (2018), who speculated that the icosalide-positive *Aureobasidium* culture may have contained an associated bacterial icosalide producer in or on the hyphae. Nonetheless, the structures of the icosalides (analogues of A1, A2 and B) were described as containing a peptide moiety of Leu-Ser-Ser-Leu (varying in D/L-configuration) attached to two fatty acid chains (that may vary in length of C_8_ and C_10_) (Figure 2) (Dose et al., 2018; Jenner et al., 2019). Boros et al. (2006) then found that icosalide A1 exhibits promising antibacterial activity against *Streptococcus pyogenes* (MICs of 8–16 μg/ml). In addition, icosalide A1 displayed activity against *Bacillus thuringensis* (MIC of 12.5 μg/ml) and *E. faecium* (MIC of 16 μg/ml) (Dose et al., 2018; Jenner et al., 2019).

Malleipeptins are partially cyclised lipopeptides produced by *B. pseudomallei* strains (Biggins et al., 2014). Malleipeptin A is comprised of a peptide moiety of 12 amino acids (i.e., Ser-Glu-Ser-Lys-Thr-Leu-Dab-Thr-Thr-Glu-Gly-Ile, where Dab refers to 2,4-diaminobutyric acid) cyclised between the final four amino acids, linked to a fatty acid chain (Figure 2). Malleipeptin B differs from A based on a substitution in the twelfth amino acid position (Biggins et al., 2014). The same lipopeptide was independently described by Esmaeel et al. (2016) following the exploration of the genomes of 48 *Burkholderia* strains and was subsequently named burkhomycin. Glidopeptin A is a linear lipopeptide from a *Burkholderia* DSM7029 strain with structural similarity to malleipeptin and is comprised of 12 amino acids (i.e., Ser-Glu-Dab-Lys-Dhb-Leu-Dab-Ser-Dhb-Asn-Gly-Dhv; where Dhb refers to dehydrobutyrine and Dhv refers to dehydrovaline) linked to a fatty acid chain (decanoic acid) (Figure 2) (Wang et al., 2018). Weak inhibition activities (100 μg/ml) were observed for glidopeptin A against *S. aureus* and *B. subtilis* (Wang et al., 2018), while the antimicrobial activity of malleipeptins/burkhomycin is currently unknown.

The “haereo” families of lipopeptides are comprised of the structurally similar haereogladins, haereoglumins, haereogladiodins and haereoplantins. Haereogladins and haereoglumins are linear lipopeptide families isolated from *B. gladioli* pv*. agaricicola* and *B. glumae* strains*,* respectively (Thongkongkaew et al., 2018). Haereogladin A is comprised of a peptide moiety of Dhb-Dhb-βHtyr-pHpg-PABA-Thr (where βHtyr refers to β-Hydroxytyrosine, pHpg refers to p-hydroxyphenyl glycine and PABA refers to p-amino benzoate) linked to an octanoic acid (Figure 2). Analogues (B–E) of this lipopeptide differ based on the change of βHtyr to Tyr and the presence or absence of the final amino acid (i.e., Thr) (Thongkongkaew et al., 2018). The structurally similar haereoglumin A is comprised of a peptide moiety of Dhb-Dhb-Leu-Leu-PABA-Thr linked to an octanoic acid (Figure 2). An analogue (haereoglumin B) of this lipopeptide was additionally characterised, which differed from A based on the absence of the final two residues (PABA and Thr) (Thongkongkaew et al., 2018). More recently, Chen et al. (2021) identified haereogladiodin A produced by *B. gladioli* which was comprised of a peptide moiety of Dhb-Dhb-Tyr-Leu-PABA-Thr linked to an octanoic acid, while B is comprised of Dhb-Dhb-Tyr linked to an octanoic acid (Figure 2). Structurally similar haereoplantins were then recently discovered from *B. plantarii* (Yoshimura et al., 2020). Haereoplantin A is comprised of Dhb-Dhb-(β-OH-Leu)-Hpg-PABA-Thr linked to octanoic acid. Analogues (B–H) varied in the acyl group or amino acid positions (3 or 4 or the lack of Thr) (Yoshimura et al., 2020; Li et al., 2021b). Haereogladins and haereoglumins were evaluated for biological activity against several bacterial and fungal strains, such as MRSA, VRE, *P. aeruginosa* and *C. albicans*, amongst others; however, no activity was observed (Thongkongkaew et al., 2018). Similar results were reported by Chen et al. (2021) for haereogladiodin A and B, while the antimicrobial activity of haereoplantins is unknown.

The “burrio” families of lipopeptides consists of the structurally similar burriogladins, burrioglumins, burriogladiodins and burrioplantins. Burriogladins and burrioglumins are linear lipopeptide families first isolated from *B. gladioli* pv*. agaricicola* and *B. glumae* strains*,* respectively (Thongkongkaew et al., 2018)*.* Burriogladin A is comprised of a peptide moiety of Pro-Phe-pHpg-Ala-Glu-Pro-Dhb linked to a fatty acid chain (β-hydroxydecanoic acid) (Figure 2) (Thongkongkaew et al., 2018). In comparison, burrioglumin A is comprised of a peptide moiety of Dhb-Pro-Ser-Ala-Val-Phe-Pro-Thr linked to β-hydroxydecanoic acid (Figure 2). An analogue (burrioglumin B) of this lipopeptide was additionally characterised, which differed from A based on a substitution of Val^5^ with Leu^5^. More recently, Chen et al. (2021) discovered burriogladiodins produced by *B. gladioli.* Burriogladiodin A is comprised of a peptide moiety of Dhb-Pro-Gln-Ala-Val-Phe-Pro linked to β-hydroxydecanoic acid (Figure 2), while burriogladiodin B has a peptide moiety of Dhb-Pro-Gln-Ala-Val-Phe-Pro-Thr. Additional analogues of burriogladiodin (C–H) were identified, that differ from B based on the amino acid substitutions and/or the absence of one or both final two residues (Chen et al., 2021). Burrioplantin A was also recently discovered from *B. plantarii* (Yoshimura et al., 2020), with a structure of Dhb-Pro-Ser-Ala-Hpg-Phe-Pro-Homoserine linked to β-hydroxydecanoic acid (Yoshimura et al., 2020). The antimicrobial potential of these lipopeptides is still currently unknown, despite efforts to test burriogladins and burriogladiodins against selected Gram-negative and Gram-positive bacteria (Thongkongkaew et al., 2018; Chen et al., 2021).

## Antimicrobial lipopeptides: Production by underexplored predatory bacterial genera

### Myxococcus

The Gram-negative, rod-shaped *Myxococcus* genus belongs to the Myxococcaceae family (Chambers et al., 2020), with seven validly published species identified to date (available at: https://lpsn.dsmz.de/genus/myxococcus). This genus is ubiquitous in soil environments and has been isolated from various climates, including temperate zones, tropical rain forests, arctic tundra and deserts, amongst others (Mohr, 2018). Like other myxobacteria, these predators can move in co-ordinated swarms by gliding over solid surfaces; however, under nutrient-deficient conditions, they can form fruiting bodies in which myxospores are produced (Mohr, 2018). Although *Myxococcus* species produce a wide range of secondary metabolites and hydrolytic enzymes (i.e., proteases, lipases, peptidases, and glycoside hydrolases) that assist in the lysis and degradation of their prey organisms, which include Gram-negative and Gram-positive bacteria as well as fungi, their potential lipopeptide production has been relatively underexplored (Burgard et al., 2017). Currently, one lipopeptide family has been characterised from this genus, namely the myxochromide lipopeptides (Hu et al., 2021).

**FIGURE 3:**
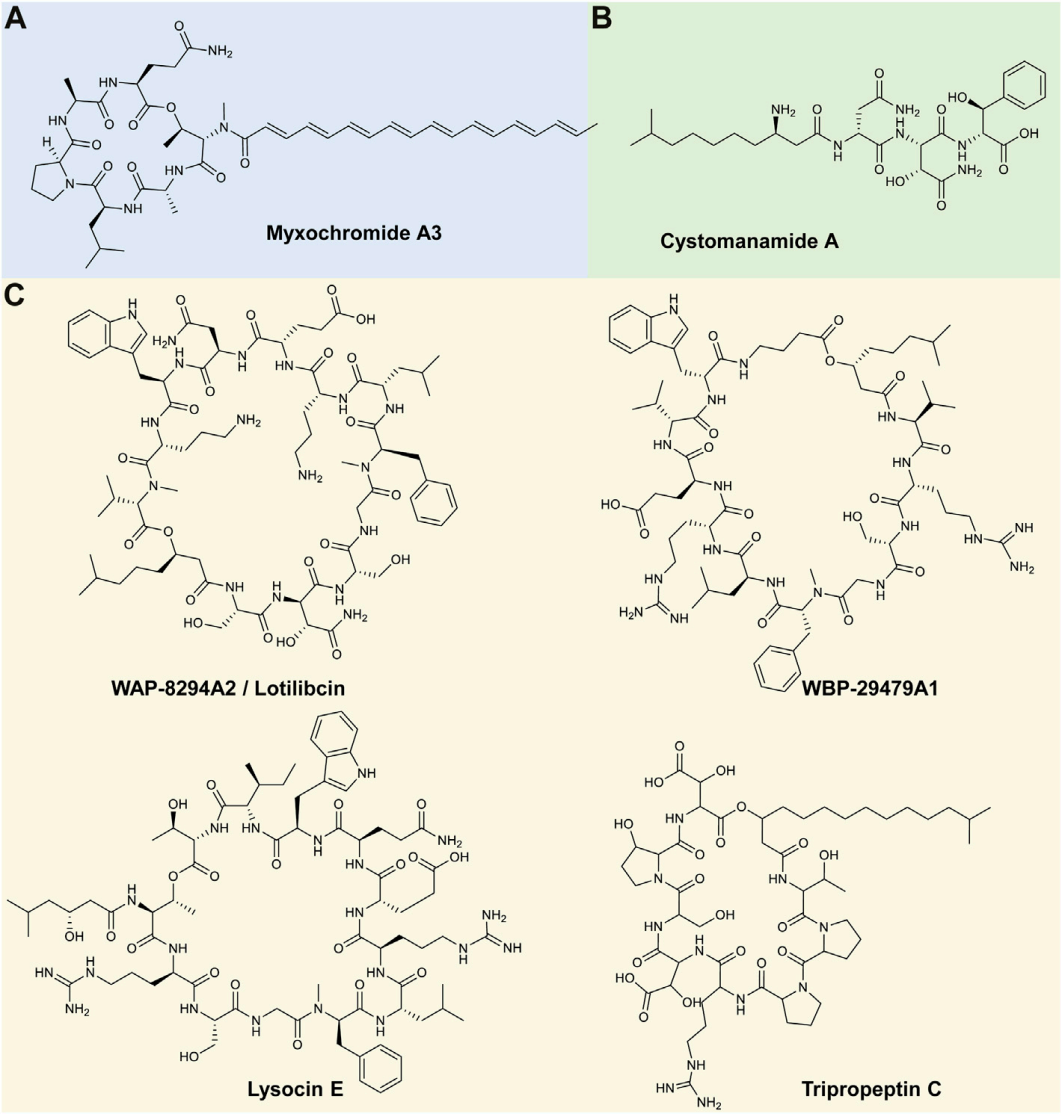
Representative lipopeptides produced by **(A)** *Myxococcus* species, **(B)** *Cystobacter* species and **(C)** *Lysobacter* species. The structural information was obtained from the PubChem database (https://pubchem.ncbi.nlm.nih.gov/), as well as the corresponding references provided in-text. The structures were subsequently drawn in ChemDraw Ultra version 12.0.

The myxochromides are a group of cyclic depsipeptides that contain an unsaturated polyketide chain (Ohlendorf et al., 2008). Myxochromides were originally described by Wenzel et al. (2005) and were produced by a myxobacterium *Stigmatella aurantiaca* strain (producing analogues of S_1-3_) and by a *M. virescens* strain (producing the myxochromide A analogue). Myxochromide A is a lipohexapeptide containing a peptide moiety of Thr-Ala-Leu-Pro-Ala-Gln linked to a heptaenoic acid (Wenzel et al., 2005). A follow up study by Wenzel et al. (2006) further identified a *M. xanthus* strain that could produce myxochromide analogues (A_2-4_), which have the same peptide core as myxochromide A but differ in the polyketide chain (Figure 3A) (Wenzel et al., 2006; Ohlendorf et al., 2008). In 2008, the myxochromide B subgroup was elucidated from a *Myxococcus* sp. strain (Ohlendorf et al., 2008), where myxochromide B_3_ was found to share a similar peptide sequence as myxochromide A; however, myxochromide B_3_ contained an additional Leu residue (Ohlendorf et al., 2008; Burgard, 2017). Burgard et al. (2017) further reported on the isolation of four novel myxochromides types, namely the C, D, S-Abu and S-di-Abu subgroups. The C subgroup of myxochromides had a similar peptide structure to the A-type myxochromides but without the Ala residue between the Pro and Glu residues, while the D and S subgroups myxochromides were produced by *Hyalangium*, *Cystobacterineae* sp. or *Stigmatella* species (Burgard et al., 2017). The myxochromide S_1-3_ and B_3_ were tested against Gram-positive (such as *S. aureus*) and Gram-negative bacteria (such as *E. coli*); however, no activity has been observed to date (Wenzel et al., 2005; Ohlendorf et al., 2008; Burgard et al., 2017).

### Cystobacter

The Gram-negative *Cystobacter* genus is comprised of seven validly published species (available at: https://lpsn.dsmz.de/genus/cystobacter) and although previous taxonomic studies have indicated that this genus belongs to the Archangiaceae family, a recent study by Waite et al. (2020) proposed its reclassification as a member of the Myxococcaceae family, within the Myxococcales order. These myxobacteria are ubiquitous in soil environments and similar to *Myxococcus* species, *Cystobacter* utilise gliding motility to move over surfaces, and are also able to form spore-filled fruiting bodies when subjected to unfavourable conditions (Akbar et al., 2017; Treuner-Lange et al., 2017). However, the *Cystobacter* fruiting bodies differ in morphology to those of the *Myxococcus* genus, and are shiny, spherical and held within a slime matrix, whereas the latter are characterised by haystack-shaped cell aggregates (Treuner-Lange et al., 2017). *Cystobacter* species have a relatively large genome size, thus enabling them to produce a vast range of secondary metabolites, including bacteriocins, polyketides, lantipeptides and microcins (Saadatpour and Mohammadipanah, 2020). Although their potential lipopeptide production has been relatively underexplored (Burgard et al., 2017), one lipopeptide family, namely the cystomanamide lipopeptides, has been characterised (Etzbach et al., 2014).

The cystomanamides are a family of four structurally similar linear lipopeptides isolated from *C. fuscus* MCy9118 (Etzbach et al., 2014). Cystomanamide A contains a peptide moiety of Asn-(β-OH-Asn)-(β-OH-Phe) linked to a fatty acid chain (i.e., 3-amino-9-methyldecanoic acid) (Figure 3B) (Etzbach, 2015). Analogues (B–D) have been identified and differ from cystomanamide A based on the addition of a Tyr and glyceric acid residues (B), fructose (C) or the addition of fructose and substitution of β-OH-Phe with Phe (D). Similar to the myxochromides produced by *Myxococcus*, to date the cystomanamides have not been reported to exhibit any antimicrobial activity (Etzbach et al., 2014; Herrmann et al., 2017).

### Lysobacter

The Gram-negative *Lysobacter* genus belong to the Xanthomonadaceae family (Christensen and Cook, 1978) and are currently comprised of 67 validly published species (available at: https://lpsn.dsmz.de/genus/lysobacter). *Lysobacter* species have frequently been detected in soil, rhizosphere and freshwater environments and their micro-predatory activity against various Gram-negative and Gram-positive bacteria, fungi, yeasts and even nematodes, has been reported (Hayward et al., 2010; De Bruijn et al., 2015). Moreover, *Lysobacter* species have been recognised as “peptide production specialists” (Panthee et al., 2016). The majority of the *Lysobacter* species have BGCs encoding for approximately 15 secondary metabolites, including NRPSs, NRPS hybrids, lantipeptides, bacteriocins, terpenes, amongst others (Panthee et al., 2016). Moreover, several lipopeptides that exhibit antimicrobial activity have been identified.

The WAP-8294A lipopeptide family was first isolated from a *Lysobacter* sp. WAP-8294 strain and numerous analogues (such as A1, A2, A4, Ax8, Ax9, and Ax13, etc.) of this lipopeptide have since been isolated from several *Lysobacter* sp. (Kato et al., 1997; Chen et al., 2019). This family of lipopeptides consists of at least 19 closely related cyclic lipodepsipeptides, where WAP-8294A2 was identified as the major compound produced (Harada et al., 2001). The WAP-8294A2 has a peptide moiety of 12 amino acids [peptide sequence of Ser-Asn-Ser-Gly-(*N*me-Phe)-Leu-Orn-Glu-Asn-Trp-Orn-(*N*meVal)], linked to a fatty acid chain (3-hydroxyl-7-methyloctanoic acid) (Figure 3C) (Zhang et al., 2011). Other members of this complex contain a similar scaffold structure but differ in their fatty acid component (Chen et al., 2019). Recently, new analogues of WAP-8294A were identified (AZ1–AZ4, AZ6, and AZ7) from a *L. enzymogenes* OH11 strain, where the structures differed from A2 in one amino acid residue or the length of the fatty acid chain (Zhu et al., 2022). Due to its potent antimicrobial activity against MRSA, WAP-8294A2 (also referred to as Lotilibcin) underwent phase I/II clinical trials by aRigen Pharmaceuticals in 2009 (Gómez Expósito et al., 2015; Yue et al., 2022). Currently, stage II clinical trials, for the topical application treatment of Gram-positive bacterial infections, are underway (Dijksteel et al., 2021).

A new cyclic lipopeptide, named WBP-29479A1, was discovered utilising genome mining analysis and was isolated from *L. antibioticus* ATCC 29479 (Sang et al., 2019). The peptide structure of this compound is comprised of Val-Arg-Ser-Gly-(*N*me-Phe)-Leu-Arg-Glu-Val-Trp-Aba (where Aba refers to aminobutyric acid) (Figure 3C). This compound also exhibited strong antimicrobial activity against MRSA (MICs of 0.25–2 μg/ml) and *Staphylococcus epidermis* (MIC ≤0.25 μg/ml) (Yue et al., 2022).

Lysocins were isolated from *Lysobacter* sp. RH2180-5, and currently nine congeners have been discovered (Hamamoto et al., 2015; Panthee et al., 2016). The primary cyclic lipodepsipeptide identified in this family was named lysocin E and has a peptide moiety of 12 amino acid residues [Thr-Arg-Ser-Gly-(*N*me-Phe)-Leu-Arg-Glu-Gln-Trp-Ile-Thr], with an ester linkage to the fatty acid chain (3-OH-5-Me-hexanoic acid) (Figure 3C) (Hamamoto et al., 2015). Although similar in their amino acid sequence to other cyclic lipopeptides such as WAP-8294A2 and WBP-29479A1, the fatty acid of the lysocins does not form part of the cyclic system (Yue et al., 2022). Derivatives of lysocins (A–I) have been shown to differ in the length of the fatty acid chain, and in the methylation of Phe in the fifth position Panthee et al. (2016). While lysocin E contains Ile in the 11^th^ position, lysocin B contains Val in this position (Panthee et al., 2016). Lysocin E has exhibited the most potent antimicrobial activity, specifically against MRSA, where an MIC of 4 μg/mL has been reported. However, activity has also been reported against methicillin-resistant and methicillin-susceptible *Staphylococcus simulans*, *Staphylococcus haemolyticus* and *Staphylococcus pseudintermedius*, as well as *B. subtilis*, *Bacillus cereus* and *Listeria monocytogenes* (*L. monocytogenes*) (Hamamoto et al., 2015).

The tripropeptins are a family of lipopeptide antibiotics that were first isolated from *Lysobacter* sp. strain BMK333-48F3. One of the major components identified from this strain was tripropeptin C (TPPC) that has a structure of eight amino acids [peptide sequence of (*allo*-Thr)-Pro-Pro-Arg-(*threo*-β-OHAsp)-Ser-(*trans*-3-OH-Pro)-(threo-β-OHAsp)] linked to a fatty acid chain (13-methyl-3-hydroxy-tetrade-canoic acid) (Figure 3C) (Hashizume et al., 2011). Several derivatives of tripropeptin have been identified, each differing in the length of the fatty acid chain (Hashizume et al., 2011). Tripropeptin C is reported to exhibit the most potent antimicrobial activity against MRSA, penicillin-resistant *Streptococcus pneumoniae* and VRE (Arlt et al., 2021; Yue et al., 2022).

## Mechanism of action

The mode of action of numerous lipopeptides is to primarily target the cell membrane of bacteria and fungi (Balleza et al., 2019). Various factors, such as electrostatic interactions and hydrophobicity, play an important role in the interaction of the lipopeptide with the cell membrane, and subsequent lipopeptide insertion into the membrane bilayer (Balleza et al., 2019). This results in membrane disruption (caused by pore formation or membrane solubilisation) and affects the barrier properties of the membrane (such as ion leakage), leading to cell death (Balleza et al., 2019). In the current review, despite the promising antimicrobial activity exhibited by several *Burkholderia, Myxococcus* and *Cysobacter* lipopeptides, the mode of action of these lipopeptides has not been extensively explored. Similarily, while serrawettin W2 analogues and stephensiolides produced by *Serratia* species exhibit potent antimicrobial activity, the mode of action of these lipopeptides is currently unknown. Moreover, while Deol et al. (1973) found that 10 μg/ml of serratamolide A produced by *Serratia* species increased the rate of movement of K^+^ and H^+^ ions across the cell membrane of *S. aureus*, with no membrane permeability or cell growth inhibition detected at this lipopeptide concentration; limited information exists on the mode of action of serratamolides. Contrastingly, the mode of action of lipopeptides produced by *Brevibacillus* and *Lysobacter* species has been investigated and will be discussed in this review.

With the exception of tauramamides, the antimicrobial mechanisms of action of several lipopeptides produced by *Brevibacillus* species have been described. For example, Li et al. (2020a) investigated the mode of action of bogorol K against *S. aureus* and *X. campestris*. This lipopeptide was then found to target the cell membrane of both Gram-negative and Gram-positive bacteria by forming pores within the membrane and causing the leakage of intracellular material. In contrast, brevibacillin first binds to the lipoteichoic acid (LTA) of *S. aureus* and lipopolysaccharides (LPS) of *Salmonella typhimurium* before interacting with the cell membrane and increasing membrane permeability, resulting in the leakage of intracellular material and leading to cell death (Yang et al., 2017; Wu et al., 2021). The mode of action of brevilaterin B was also investigated and it was found to cause membrane depolarisation of *L. monocytogenes,* while liposome analysis (model membranes) confirmed that brevilaterin B integrates into the lipid bilayer and increases membrane permeability, causing leakage of intracellular material (Liu et al., 2020). Simiarly, mode of action studies revealed that brevicidine B increases membrane permeability of *S. aureus*, while causing membrane depolarisation (interfering with the proton motive force) in *E. coli* (Zhao and Kuipers, 2021). In comparison, relacidine B was found to bind to the LPS of *X. campestris* but was unable to form pores in the membrane (Li et al., 2020b). Rather, it affected the oxidative phosphorylation process of cells and diminished ATP biosynthesis (Li et al., 2020b). The laterocidines were similarly found to associate with the LPS, while no significant membrane disruption was observed against *E. coli* (Li et al., 2018). Li and co-authors (2018) thus suggested that this lipopeptide could have multiple targets and recommended additional in-depth mechanism studies.

The mechanism of action of lipopeptides produced by *Lysobacter* species, including WAP-8294A2, WBP-29479A1, lysocin E and TPPC, has been determined. Specifically, the mode of action of WAP-8294A2, WBP-2947A1 and lysocin E was revealed to cause bacterial cell membrane disruption in a menaquinone-dependent manner (Hamamoto et al., 2015; Itoh et al., 2018; Sang et al., 2019). Menaquinone is an essential component involved in electron transfer within the respiratory chain. In comparison, the potent antimicrobial activity of TPPC has been attributed to the inhibition of phosphatase activity, which is crucial for the peptidoglycan biosynthesis process. More specifically, TPPC forms a complex with undecaprenyl pyrophosphate in the presence of calcium ions and, thus interfering with the lipid cycle of the cell wall synthesis (Hashizume et al., 2011). It has also been reported that TPPC is able to potentiate and re-sensitise MRSA to beta (β)-lactam antibiotics, further indicating this compound’s promise as an effective antimicrobial agent (Hashizume et al., 2015).

## Biosynthesis of lipopeptides

Generally, lipopeptides are biosynthesised via enzymatic machinery encoded by a hybrid PKS and NRPS system (Kunakom and Eustáquio, 2019). The NRPS and PKS megaenzymes are arranged in an assembly line, where a set of catalytic domains are grouped into modules that incorporate monomers through sequential condensation (Burgard, 2017; Kunakom and Eustáquio, 2019). The PKSs are derived from fatty acid synthases and use a wide range of starter and extender units for fatty acid biosynthesis, which originate from the primary carbon metabolism (Burgard, 2017; Kunakom and Eustáquio, 2019). In general, fatty acids are synthesised via decarboxylative Claisen-type condensation of short-chain acetyl-CoA (most common starter unit) and are typically extended with malonyl-CoA or methylmalonyl-CoA (Burgard et al., 2017; Kunakom and Eustáquio, 2019). The acyl-transferase (AT) is responsible for the selection and transfer of the acetyl-CoA starter unit and extender unit (i.e., malonyl-CoA) to the acyl-carrier protein (ACP). The acetyl-CoA bound to ACP is then elongated by β-ketoacylsynthase (KS), which catalyses decarboxylative Claisen-type condensations to form C-C bonds between the starter and extender units, resulting in a β-ketoacyl still bound to the ACP. The growing ACP-bound acyl chain can then be processed by a set of reductive enzymes, including a ketoreductase (KR), a dehydratase (DH) and an enoylreductase (ER). The KR can catalyse the conversion of β-ketoacyl functionality to β-hydroxy compounds. Thereafter, the DH can catalyse the loss of water from β-hydroxy compounds and result in the formation of a C=C bond in the growing fatty acid chain (β-enoyl compounds). Finally, the β-enoyl compounds can be reduced to a saturated C-C bond by ERs, resulting in the formation of an acyl chain (C_n+2_) still bound to the ACP. This acyl-ACP can then serve as a substrate for further elongation by the addition of an extender unit via KS, resulting in long fatty acid chains of typically C_6_-C_18_ in length (Burgard, 2017; Herbst et al., 2018; Théatre et al., 2021). The long chain fatty acid-ACP is produced after additional cycles, and fatty acid precursors are released from ACP. Additional modifications may then occur, such as the transamination (addition of amino groups to the keto-acid) of the fatty acyl chain by the aminotransferases (AmT) (Théatre et al., 2021). The fatty acids are then transferred onto the peptidyl carrier protein (PCP) and condensed with the starting amino acid through the condensation (C) domain in the first NRPS module (Kraas et al., 2010; Yang et al., 2020).

The NPRSs are thus multimodular enzymes that consist of repeated modules that catalyse the synthesis of peptide products from both proteinogenic and non-proteinogenic amino acids (Miller and Gulick, 2016; Kunakom and Eustáquio, 2019). A single module is comprised of a set of conserved catalytic domains responsible for incorporating a single residue into the peptide backbone (Miller and Gulick, 2016). The adenylation (A) domain typically recognises, selects and activates (using ATP) a specific amino acid to form an aminoacyl-adenylate intermediate (Kunakom and Eustáquio, 2019). The aminoacyl-adenylate intermediate is then covalently attached to a 4′-phosphopanthetheine prosthetic group on the thiolation (T) domain or PCP via a thioester linkage. Hereafter, the PCP delivers the amino acid intermediate to the adjacent C domain to form a peptide (amine) bond to the growing peptide chain (Baltz et al., 2005; Kunakom and Eustáquio, 2019). Finally, the lipopeptide is synthesised as a linear molecule, which can be cyclised by the thioesterase (TE) domain at the end of the assembly line (in the final module within the BGC) (Baltz et al., 2005). Additional domains, such as the epimerase (E) domain, may be present in some modules to affect the conversion of L-to D-stereochemistry (Baltz et al., 2005). The order of modules is generally co-linear to the peptide sequence of the lipopeptide that is produced and the BGC can thus be used to predict the putative structure of the product (Baltz et al., 2005). Biosynthetic web-based tools, such as antiSMASH, have subsequently been developed to aid in the detection and annotation of BGCs and predict the structures of the PKS-NRPS products (Kunakom and Eustáquio, 2019).

## Genome mining for prospective lipopeptides

The NCBI has an abundance of genome sequences available (at: https://www.ncbi.nlm.nih.gov/genome/) within its database that can be used in combination with antiSMASH to determine the potential of a strain to produce various natural products, and in particular, putatively predict lipopeptides. In this review, genome data available (a total of 378 bacterial genomes) on NCBI for members of the *Serratia* (*n* = 197 strains), *Brevibacillus* (*n* = 24 strains), *Burkholderia* (*n* = 116 strains), *Myxococcus* (*n* = 16 strains), *Cystobacter* (*n* = 2 strains) and *Lysobacter* (*n* = 23 strains) genera was analysed using antiSMASH as a prediction tool to identify typical PKS-NRPS BGCs (i.e., putatively predicted to encode for lipopeptides). It is important to note that further production and structural elucidation of the natural products encoded by the newly identified BGCs is required to confirm that the hybrid PKS-NRPS clusters produce lipopeptides, and thus only putative identification and predictions of novel BGCs will be made in this review.

### Serratia

Genome mining revealed that 16 different *Serratia* species had at least one BGC encoding for PKS-NRPSs (Supplementary Table S1), while no BGC encoding for PKS-NRPSs was observed in *Serratia odorifera* (*n* = 1) or *Serratia entomophila* (*n* = 1). Moreover, the biosynthesis of serratamolides, serrawettin W2 and stephensiolides by *Serratia* species has been previously elucidated (Li et al., 2005; Su et al., 2016; Ganley et al., 2018). The gene cluster involved in the biosynthesis of serratamolides contains a PKS region (incorporating a fatty acid chain) and one NRPS-related gene with one module (predicted peptide sequence of Ser-TE) and was named *swrW* (Table 1) (Li et al., 2005; Clements et al., 2019). A serratamic acid (one fatty acid and one Ser) is formed and transferred to the TE domain, whereafter a second serratamic acid binds to the free T domain. The two neighbouring serratamic acids form an intramolecular linkage and is cyclised to form serratamolides (i.e., two fatty acids and two Ser residues) (Li et al., 2005; Clements et al., 2019). In this review, this BGC was detected in genomes from 15 different *Serratia* species, such as *S. marcescens*, *Serratia rhizosphaerae* and *Serratia bockelmannii* (*S. bockelmannii*), amongst others (Supplementary Table S1).

**TABLE 1:** Summary of the known PKS-NRPS BGCs identified from *Serratia*, *Brevibacillus,* and *Burkholderia* species.

Lipopeptide	Producing genus	Gene name	No. of NRPS modules	NRPS domains	Peptide sequence	Reference(s)
Serrawettin W1	*Serratia*	*swrW*	1	C_1_-A_1_-PCP_1_-TE	Ser	Li et al. (2005)
Serrawettin W2	*Serratia*	*swrA*	5	(C-A-PCP)_1-5_-TE	Leu-Ser-Thr-Phe-X	Su et al. (2016), Clements-Decker et al. (2022)
Stephensiolide	*Serratia*	*sphA*	5	(C-A-PCP)_1-5_-TE	Thr-Ser-Ser-Val-Ile/Val	Ganley et al. (2018)
Tauramamide	*Brevibacillus*	*tau*	5	(C-A-PCP-E)_1_-(C-A-PCP)_2_-(C-A-PCP-E)_3_-(C-A-PCP)_4-5_-TE	Phe/Trp-Ser-Leu-Phe/Trp-Phe/Arg	Dejong et al. (2016)
Bogorol	*Brevibacillus*	*bogA-F; bogJ*	13	(C-A-PCP)_1-2_-(C-A-PCP-E)_3_-(C-A-PCP)_4-6_-(C-A-PCP-E)_7_-(C-A-PCP)_8–10_-(C-A-PCP-E)_11_-(C-A-PCP)_12–13_-TD-TE	Dhb-Leu/Val-Orn/Lys-Ile/Val-Val/Ile-Val-Lys-Val-Leu-Lys-Tyr-Leu-Val	Dejong et al. (2016), Li et al. (2020a)
Bogorol-like lipopeptide	*Brevibacillus*	Not provided	13	Domain information not provided	Dhb-Tyr-Orn-Ile-Val-Val-Lys-Val-Leu-Asp-Val-Glu	Singh et al. (2021)
Brevibacillin	*Brevibacillus*	*brvA-E*	13	(C-A-PCP)_1-2_-(C-A-PCP-E)_3_-(C-A-PCP)_4-6_-(C-A-PCP-E)_7_-(C-A-PCP)_8–10_-(C-A-PCP-E)_11_-(C-A-PCP)_12–13_-TD-TE	Dhb-Leu-Orn-Val/Ile-Val/Ile-Val-Lys-Val/Ile-Val-Lys-Tyr-Leu-Val	Zhao et al. (2021)
Brevilaterin	*Brevibacillus*	*bre260-261*; *bre265-270*	13	(C-A-PCP)_1-2_-(C-A-PCP-E)_3_-(C-A-PCP)_4-6_-(C-A-PCP-E)_7_-(C-A-PCP)_8_-(C-A-PCP-E)_9_-(C-A-PCP)_10_-(C-A-PCP-E)_11_-(C-A-PCP)_12–13_-TD-TE	Thr-Leu/Met/Val/Ile-Orn/Lys-Ile/Val/Leu-Val/Ile-Val/Ile-Lys/Orn-Val/Ile-Val/Leu-Lys/Orn-Tyr/Val/Ile-Leu/Val-Val	Han et al. (2022)
Brevicidine	*Brevibacillus*	*breA-E*	12	(C-A-PCP-E)_1-4_-(C-A-PCP)_5-6_-(C-A-PCP-E)_7_-(C-A-PCP)_8–12_-TE	Asn-Phe/Tyr-Tyr/Trp-Orn-Orn-Gly-Orn-Tyr/Trp-Thr-Ile-Gly-Ser	Li et al. (2018), Zhao and Kuipers (2021)
Laterocidine	*Brevibacillus*	*latA-E*	13	(C-A-PCP-E)_1-4_-(C-A-PCP)_5-6_-(C-A-PCP-E)_7_-(C-A-PCP)_8–13_-TE	Ser-Phe/Tyr-Tyr/Trp-Orn-Orn-Gly-Orn-Tyr-Thr-Ile-Asn-Gly-Gly	Li et al. (2018)
Relacidine	*Brevibacillus*	*rlcA-E*	13	(C-A-PCP-E)_1-4_-(C-A-PCP)_5-7_-(C-A-PCP-E)_8-9_-(C-A-PCP)_10–13_-TE	Ser-Phe-Tyr-Orn-Orn-Gly-Orn-Tyr-Thr-Ile-Gly-Ser-Gly	Li et al. (2020b)
Icosalide	*Burkholderia*	*icoS*	4	(C-A-PCP)_1-2_-(C-C-A-PCP)_3_-(C-A-PCP)_4_-TE	Leu-Ser-Ser-Leu	Dose et al. (2018), Jenner et al. (2019)
Malleipeptin/Burkhomycin	*Burkholderia*	*mpnB-E*	12	(C-A-PCP)_1–12_-TE_1_-TE_2_	Ser-Glu-Ser-Lys-Thr/Dhb-Leu-Dab-Thr-Thr-Glu/hGlu-Gly-Val/Ile	Biggins et al. (2014)
Glidopeptin	*Burkholderia*	*glpA-H*	12	(C-A-PCP)_1–12_-TE_1_-TE_2_	Ser-Glu-Dab-Lys-Thr/Dab-Leu-Dab-Ser-Thr/Dhb-Asn-Gly-Val/Dhv	Wang et al. (2018)
Holrhizin	*Burkholderia*	*holA*	6	(C-A-PCP)_1-6_-TE	Val-Phe-Glu-Ile-Ala-Ile	Niehs et al. (2018)
Haereogladin	*Burkholderia*	*hgdA*	5/6	(C-A-PCP)_1–5/6_-TE	Dhb-Dhb-(β-Htyr/Tyr)-pHpg-PABA-Thr/H_2_O	Thongkongkaew et al. (2018)
Haereoglumin	*Burkholderia*	*hgmC*	5/6	(C-A-PCP)_1–5/6_-TE	Dhb-Dhb-Leu-Leu-PABA-Thr/H_2_O	Thongkongkaew et al. (2018)
Haereogladiodin	*Burkholderia*	*hgddC*	5/6	(C-A-PCP)_1–5/6_-TE	Dhb-Dhb-Tyr-Leu-PABA-Thr/H_2_O	Chen et al. (2021)
Haereoplantin	*Burkholderia*	*hptC*	5/6	(C-A-PCP)_1-3_-(C-A-PCP-E)_4_-(C-A-PCP)_5/6_-TE	Dhb-Dhb-(β-OH-Leu)-Hpg-PABA-Thr	Yoshimura et al. (2020)
Burriogladin	*Burkholderia*	*bgdA-B*	6/7	(C-A-PCP)_1–6/7_-TE	Dhb-Pro-Glu-Ala-pHpg-Phe-Pro-Thr/H_2_O	Thongkongkaew et al. (2018)
Burrioglumin	*Burkholderia*	*bgmA*	7	(C-A-PCP)_1-7_-TE	Pro-Ser-Ala-Val/Leu-Phe-Pro-Thr	Thongkongkaew et al. (2018)
Burriogladiodin	*Burkholderia*	*bgddA*	7/8	(C-A-PCP)_1–7/8_-TE	Dhb-Pro-Glu-Ala-Val/Ala/Ile/Leu-Phe-Pro-Thr/H_2_O	Chen et al. (2021)
Burrioplantin	*Burkholderia*	*bptE*	7/8	(C-A-PCP)_1_-(C-A-PCP-E)_2-4_-(C-A-PCP)_5_-(C-A-PCP-E)_6_-(C-A-PCP)_7/8_-TE	Dhb-Pro-Ser-Ala-pHpg-Phe-Pro-Homoserine	Yoshimura et al. (2020)

TD, terminal reductase.

The gene cluster involved in the biosynthesis of serrawettin W2 contains a PKS region, named *swrEFG* (incorporates a fatty acyl chain), and one NRPS-related gene, namely *swrA,* with five modules (predicted peptide sequence of Leu-Ser-Thr-Phe-X-TE, where X represents a position with no reliable prediction) (Table 1) (Su et al., 2016; Clements-Decker et al., 2022). The varying amino acid moiety of the elucidated serrawettin W2 analogues suggests that the A domain of modules 4 and 5 in *swrA* have reduced amino acid specificity (Clements-Decker et al., 2022). This BGC was detected in the genomes of strains from three different *Serratia* species in this review, including *S. marcescens*, *S. surfactantfaciens* and *S. ureilytica* (Supplementary Table S1). Additionally, the gene cluster involved in the biosynthesis of stephensiolides contains a PKS region incorporating one fatty acyl chain and one NRPS gene, named *sphA,* with five modules (predicted peptide sequence of Thr-Ser-Ser-Val-Val/Ile-TE) (Supplementary Table S1) (Ganley et al., 2018). Similar to *swrA*, the varying amino acid moiety of the elucidated stephensiolide analogues suggests that the A domain of modules 4 and 5 in *sphA* have reduced amino acid specificity. This BGC was detected in the genomes of strains from four different *Serratia* species analysed in this review, including *S. fonticola, Serratia ficaria* (*S. ficaria*)*, S. marcescens* and *S. ureilytica* (Supplementary Table S1).

**FIGURE 4:**
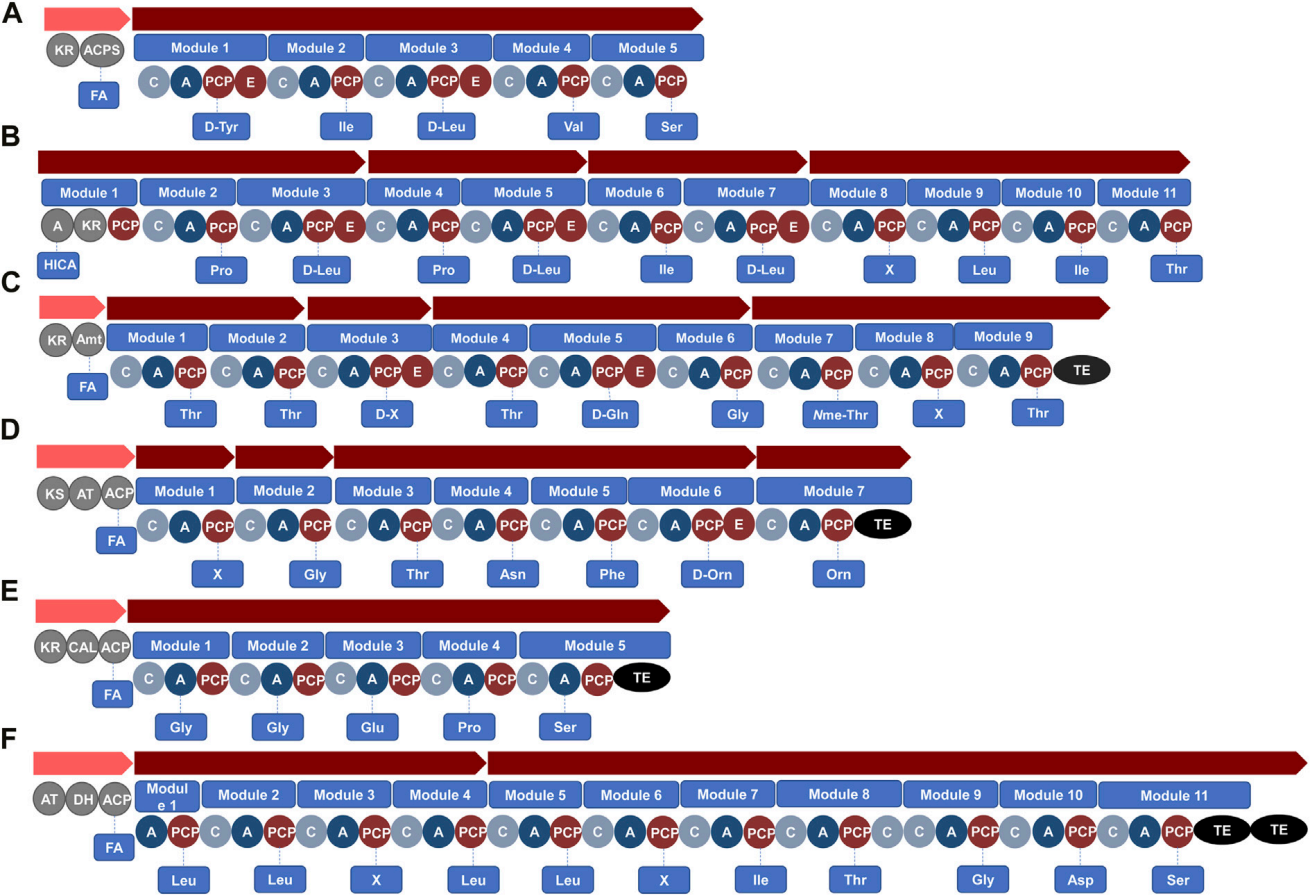
Unknown bacterial BGCs identified using antiSMASH in the genomes of **(A)** *S. fonticola* R28 (NCBI accession no. CP072742.1), **(B)** *B. laterosporus* LMG 15441 (NCBI accession no. CP007806.1), **(C)** *B. mayonis* BDU8 (NCBI accession no. CP013389.1), **(D)** *M. xanthus* GH5.1.9c20 (NCBI accession no. CP017170.1), **(E)** *C. fuscus* DSM 52655 (NCBI accession no. CP022098.1) and **(F)** *L. gummosus* 10.1.1 (NCBI accession no. CP093547.1). Domains within modules of the gene clusters that encode for PKS or NRPS enzymes include: KR, ketoreductase; KS, ketoacylsynthase; DH, dehydratase; AT, acyl-transferase; ACPS, phosphopantetheinyl transferase; C, condensation; A, adenylation; PCP, peptidyl carrier protein; E, epimerisation; Amt, aminotransferases; ER, enoylreductase; TE, thioesterase. Abbreviations: FA, fatty acid; HICA, α-hydroxy-isocaproic-acid; X, unknown predicted amino acid.

A total of 18 unknown BGCs, with a hybrid PKS-NRPS system that did not correspond to previously characterised lipopeptide gene clusters, were then identified within *Serratia* species using genome mining. For instance, antiSMASH revealed a BGC that putatively encodes for a novel linear (no TE domain in the BGC) lipopeptide with the structure FA-Tyr-Ile-Leu-Val-Ser that was detected within genomes of *S. fonticola* (*n* = 6 strains) (Figure 4A; Supplementary Table S1), while the same gene (without the PKSs incorporating a fatty acid) was also detected in a *S. bockelmannii* strain (Supplementary Table S1). Moreover, antiSMASH analysis conducted in this review putatively identified 12 species (such as *S. brockelmannii*, *S. ficaria*, *S. fonticola*, *Serratia grimesii*, *Serratia inhibens* and *Serratia symbiotica*) as new lipopeptide producers (not previously reported to produce lipopeptides) as they contain at least one BGC that putatively encodes for a lipopeptide (hybrid PKS-NRPS system) (Supplementary Table S1).

### Brevibacillus

Genome mining conducted for this review revealed that six different *Brevibacillus* species had at least one BGC encoding for PKS-NRPSs (Supplementary Table S2), while no BGC encoding for PKS-NRPSs was observed in *Brevibacillus choshinensis* (*n* = 1) and *Brevibacillus marinus* (*n* = 1). Moreover, the biosynthesis of tauramamide, bogorol, brevibacillin, brevilaterin, brevicidine, laterocidine and relacidine by *Brevibacillus* species has previously been elucidated (Table 1). The gene cluster involved in the biosynthesis of tauramamide contains a PKS region and one NRPS gene of five modules (predicted peptide sequence of Phe-Ser-Leu-Trp-Arg-TE) and was named *tau* (Table 1) (Desjardine et al., 2007; Dejong et al., 2016). The varying amino acid moiety of the elucidated tauramamide analogues suggests that the A domain of modules 1, 4 and 5 in *tau* have reduced amino acid specificity (Dejong et al., 2016). This BGC has been detected in *B. laterosporus* strains (Dejong et al., 2016) and was similarly detected in this species during genome mining conducted for this review (Supplementary Table S2).

Similar gene clusters have previously been identified for the synthesis of bogorol, brevibacillin and brevilaterin (Li et al., 2020a). The BGCs contain a PKS region and five NRPS-related genes in the BGCs of bogorol (named *bogA* - *bogF*) and brevilaterin (named *bre265*–*bre270*), while brevibacillin has four NRPS-related genes (named *brvB–brvE*). All three NRPS-related genes contain 13 modules, with the predicted peptide sequence for bogorol as Dhb-Leu/Val-Orn/Lys-Ile/Val-Val/Ile-Val-Lys-Val-Leu-Lys-Tyr-Leu-Val, brevilaterin as Dhb-Leu-Orn-Val/Ile-Val/Ile-Val-Lys-Val/Ile-Val-Lys-Tyr-Leu-Val, and for brevibacillin as Dhb-Leu-Orn-X-X-Val-Lys-X-Val-Lys-Tyr-Leu-Val, where the final amino acid (Val) is converted into valinol (Table 1) (Li et al., 2020a). For bogorol, variation in the peptide moiety suggests that the A domain in modules 2 – 5 and 9 had reduced specificity (Li et al., 2020a). Similarly, the A domain in modules 2, 3, 4, and 6 for brevilaterin and modules 4, 5 and 8 for brevibacillin likely have reduced amino acid specificity (Ning et al., 2021; Zhao et al., 2021; Chen et al., 2022). These three BGC have been detected in *B. laterosporus* strains, while only the bogorol BGC was detected in *B. laterosporus* strains using the genome mining analysis conducted for this review (Supplementary Table S2).

The gene cluster involved in the biosynthesis of brevicidine contains a PKS region and two NRPS-related genes of 12 modules (named *breC–breD*) with a predicted peptide sequence of Asn-X-Tyr-Orn-Orn-Gly-Orn-Tyr-Thr-Ile-Gly-Ser (Table 1) (Li et al., 2018). The variation in the peptide moiety of brevicidine suggests that the A domain in module 2 has reduced amino acid specificity (Zhao and Kuipers, 2021). The brevicidine gene cluster has been detected in *B. laterosporus* strains (Li et al., 2018) and was similarly detected in strains of this species using the genome mining analysis (of the 24 *Brevibacillus* strains available on NCBI) (Supplementary Table S2).

Similar gene clusters have previously been identified for the biosynthesis of laterocidine and relacidine (Li et al., 2020b). A PKS region and two NRPS-related genes were detected in the BGCs of laterocidine (named *latC–latD*) and relacidine (named *rlcC* - *rlcD*). Both BGCs contain 13 modules, with a predicted peptide sequence for laterocidine as Ser-Tyr-Trp-Orn-Orn-Gly-Orn-Trp-Thr-Ile-Asn-Gly-Gly and relacidine as Ser-Tyr-Trp-Orn-Orn-Gly-Orn-Trp-Thr-Ile-Gly-Ser-Gly (Table 1) (Li et al., 2020b). The variation in the peptide moiety of relacidine suggests that the A domain in module 13 has reduced amino acid specificity (Li et al., 2020b), while no variation in the peptide moiety of laterocidine has been described. Both BGCs have been detected in *B. laterosporus* strains (Li et al., 2020b) and were similarly detected in strains of this species using the genome mining analysis conducted (Supplementary Table S2).

It was, however, interesting to note that a total of 9 unknown BGCs with a hybrid PKS-NRPS system, were identified in *Brevibacillus* species through genome mining, which did not correspond to previously characterised lipopeptides. For instance, antiSMASH conducted in this review revealed a BGC that putatively encodes for a novel linear lipopeptide with a PKS region and four NRPS-related gene clusters containing 11 modules (with a putative sequence of α-hydroxy-isocaproic-acid-Pro-Leu-Pro-Leu-Ile-Leu-X-Leu-Ile-Thr, with variation in the module 8) (Figure 4B), with this BGC detected within genomes of *B. laterosporus* strains (Supplementary Table S2). Moreover, antiSMASH analysis putatively identified four species (i.e., *Brevibacillus agri*, *Brevibacillus composti*, *Brevibacillus formosus* and *Brevibacillus parabrevis*) as new lipopeptide producers containing at least one BGC that putatively encodes for a lipopeptide (hybrid PKS-NRPS system) (Supplementary Table S2).

### Burkholderia

Genome mining conducted for this review revealed that 21 different *Burkholderia* species had at least one BGC encoding for PKS-NRPSs (Supplementary Table S3), while no BGC encoding for PKS-NRPSs was observed in *B. humptydooensis* (*n* = 2), *B. anthina* (*n* = 3), *B. dolosa* (*n* = 6), *B. territorii* (*n* = 2), *B. diiffusa* (*n* = 1), *B. pseudomultivorans* (*n* = 1), *B. aenigmatica* (*n* = 1), *B. latens* (*n* = 3), *B. multivorans* (*n* = 9) and *B. metallica* (*n* = 1). Moreover, the biosynthesis of icosalides, malleipeptin/burkhomycin, glidopeptin, haereogladin, burriogladin, haereoglumins, burrioglumins, haereogladiodins and burriogladiodin A has been elucidated in literature (Table 1). Although the BGC for burkholdines was identified, the biosynthetic pathway was not described (Mullins et al., 2019).

The gene cluster involved in the biosynthesis of icosalides has previously been described to contain two PKS regions (incorporates two fatty acids) and one NRPS-related gene of four modules (with a predicted peptide sequence of Leu-Ser-Ser-Leu-TE), which was named *icoS* (Table 1) (Dose et al., 2018; Jenner et al., 2019). The icosalide gene cluster has been detected in *B. gladioli* strains (Dose et al., 2018; Jenner et al., 2019), while the genome mining analysis (of the 116 *Burkholderia* strains available on NCBI) conducted for this review detected the gene cluster in *B. gladioli* and *Burkholderia stagnalis* (*B. stagnalis*) strains (Supplementary Table S3).

Similar gene clusters have previously been identified for the synthesis of malleipeptin/burkhomycin and glidopeptin (Biggins et al., 2014; Wang et al., 2018). The BGCs contain a PKS region and three NRPS-related genes in the BGCs of malleipeptin/burkhomycin (named *mpnB*–*mpnD*) and glidopeptin (named *glpC*–*glpE*). Both BGCs contain 12 modules, with a predicted peptide sequence of malleipeptin as Ser-Glu-Ser-Lys-Thr-Leu-Dab-Thr-Thr-Glu-Gly-X and glidopeptin as Ser-Glu-Dab-Lys-X-Leu-Dab-Ser-X-Asn-Gly-X (Table 1) (Biggins et al., 2014; Wang et al., 2018). The variation in the peptide moiety of malleipeptin/burkhomycin suggests that the A domain in module 12 has reduced specificity (Wang et al., 2018). The variation in the peptide moiety of glidopeptin suggests that the A domain in modules 5, 9, and 12 has reduced amino acid specificity (Biggins et al., 2014). The malleipeptin/burkhomycin BGC has been detected in *B. pseudomallei*, *B. mallei* and *B. thailandensis* strains (Biggins et al., 2014), while the genome mining analysis conducted for this review detected the malleipeptin/burkhomycin gene cluster in *B. pseudomallei* (Supplementary Table S3). In comparison, the glidopeptin BGC has been detected in a *Burkholderia* DSM7029 strain (Wang et al., 2018).

Similar gene clusters have previously been identified for the synthesis of haereogladin, haereoglumins and haereogladiodins (Thongkongkaew et al., 2018; Chen et al., 2021). The BGCs contain a PKS region and one NRPS-related gene in the BGCs of haereogladin (named *hgdA*), haereoglumins (named *hgmC*) and haereogladiodins (named *hgddC*) (Table 1). All three BGCs contain 6 modules, with predicted peptide sequences of haereogladin as Dhb-Dhb-(β-Htyr/Tyr)-pHpg-PABA-Thr/H_2_O, haereoglumins as Dhb-Dhb-Leu-Leu-PABA-Thr/H_2_O, and haereogladiodins as Dhb-Dhb-Tyr-Leu-PABA-Thr/H_2_O (Thongkongkaew et al., 2018; Chen et al., 2021). The variation in the peptide moiety of haereogladin suggests that the A domain in module 4 and 6, modules 4 – 6 for haereogladiodins, and modules 5 and 6 for haereoglumins have reduced amino acid specificity (Thongkongkaew et al., 2018; Chen et al., 2021). The haereogladin, haereoglumins and haereogladiodins BGCs have been detected in *B. gladioli, B. plantarii* and/or *B. glumae* strains (Thongkongkaew et al., 2018; Chen et al., 2021), while the genome mining analysis conducted for this review detected the haereogladin gene cluster in *B. gladioli, B. plantarii* and *Burkholderia perseverans* (Supplementary Table S3).

Similar gene clusters have previously been identified for the synthesis of burriogladin, burrioglumins and burriogladiodin (Thongkongkaew et al., 2018; Chen et al., 2021). The BGCs contain a PKS region and one or two NRPS-related genes in the BGCs of burriogladin (named *bgdA* and *bgdB*), burrioglumins (named *bgmA*) and burriogladiodin (named *bgddA*) (Table 1). Despite the three BGCs differing in the number of modules, similarity was observed between the peptide sequences of Dhb-Pro-Glu-Ala-pHpg-Phe-Pro (i.e., 6 modules) for the BGC of burriogladin, Dhb-Pro-Ser-Ala-Val/Leu-Phe-Pro-Thr (i.e., 8 modules) for the BGC of burrioglumins, and Dhb-Pro-Glu-Ala-Val/Ala/Ile/Leu-Phe-Pro (i.e., 7 modules) for the BGC of burriogladiodin (Table 1) (Thongkongkaew et al., 2018). The variation in the peptide moiety of burrioglumins suggests that the A domain in module 5 and modules 5–7 for burriogladiodin exhibited reduced amino acid specificity. The haereogladin, haereoglumins and haereogladiodins BGCs have been detected in *B. gladioli, B. plantarii* and/or *B. glumae* strains (Thongkongkaew et al., 2018; Chen et al., 2021), while the genome mining analysis conducted for this review detected the burriogladin gene cluster in *B. gladioli* and burrioglumin in *B. glumae* and *B. plantarii* (Supplementary Table S3).

A total of 19 unknown BGCs with a hybrid PKS-NRPS system were identified that did not correspond to lipopeptides previously characterised in *Burkholderia* species. For instance, antiSMASH analysis conducted in this review revealed a BGC that putatively encodes for a novel cyclic lipopeptide with a PKS region and four NRPS-related gene clusters containing nine modules (with a putative sequence of FA-Thr-Thr-X-Thr-Gln-Gly-(*N*me-Thr)-X-Thr-TE, with variation in modules 3 and 8) (Figure 4C) and this BGC was detected within genomes of *Burkholderia mayonis* (*B. mayonis*)*, Burkholderia oklahomensis, B. stagnalis and Burkholderia ubonensis* (Supplementary Table S3). Moreover, antiSMASH analysis putatively identified 16 species (such as *B. cepacia, Burkholderia lata, Burkholderia pyrrocinia, Burkholderia stabilis* and *Burkholderia vietnamiensis,* etc*.*) as new lipopeptide producers containing at least one BGC that putatively encodes for a lipopeptide (hybrid PKS-NRPS system) (Supplementary Table S3).

### Myxococcus

All 16 of the *Myxococcus* genomes (four different species) from the NCBI database that were analysed using antiSMASH were found to contain at least one BGC encoding for a hybrid PKS-NRPS system (Supplementary Table S4). The BGC involved in the production of myxochromide A, B and C has previously been identified and elucidated (Table 2). The BGCs for all three contain a PKS region (named *mchA*) and two NRPS-related genes in the BGCs (named *mchB* and *mchC*). Despite the three BGCs differing in the number of modules, similarity was observed between the peptide sequences of Thr-Pro/Ala-Leu-Pro-Ala/Phe-Gln (i.e., 6 modules) for the BGC of myxochromide A, Thr-Ala/Pro-Leu-Leu-Pro-Ala/Phe-Gln (i.e., 7 modules) for the BGC of myxochromide B, and Thr-Pro/Ala-Leu-Pro-Gln (i.e., 5 modules) for the BGC of myxochromide C (Table 2) (Wenzel et al., 2005; Burgard et al., 2017; Yan et al., 2018). The variation in the peptide moiety of myxochromides suggests that the A domain in module 2 and 5 for myxochromide A, modules 2 and 6 for myxochromide B, and module 2 for myxochromide C exhibited reduced amino acid specificity. The myxochromide A, B or C BGCs have been detected in *M. xanthus, M. virescens, Myxococcus hansupus* (*M. hansupus*) and/or *Myxococcus fulvus* (Burgard et al., 2017), while genome mining analysis in this review detected the myxochromide A BGC in *M. xanthus* and myxochromide C in *M. xanthus* and *M. hansupus* (Supplementary Table S4).

**TABLE 2:** Summary of the known PKS-NRPS BGCs identified from *Myxococcus*, *Cystobacter* and *Lysobacter* species.

Lipopeptide	Producing genus	Gene name	No. of NRPS modules	NRPS domains	Peptide sequence	Reference(s)
Myxochromide A-type	*Myxococcus*	*mchA-C*	6	(C-A-MT-PCP)_1_-(C-A-PCP-E)_2_-(C-A-PCP)_3-5_-(C-A-PCP-TE)_6_	Thr-Pro/Ala-Leu-Pro-Ala/Phe-Gln	Wenzel et al. (2005), Burgard et al. (2017)
Myxochromide B-type	*Myxococcus*	*mchA-C*	7	(C-A-MT-PCP)_1_-(C-A-PCP-E)_2_-(C-A-PCP)_3-6_-(C-A-PCP-TE)_7_	Thr-Ala/Pro-Leu-Leu-Pro-Ala/Phe-Gln	Burgard et al. (2017)
Myxochromide C-type	*Myxococcus*	*mchA-C*	5	(C-A-MT-PCP)_1_-(C-A-PCP-E)_2_-(C-A-PCP)_3-4_-(C-A-PCP-TE)_5_	Thr-Pro/Ala-Leu-Pro-Gln	Burgard et al. (2017)
Cystomanamide	*Cystobacter*	*cymA-D*	5	(C-A-PCP-E)_1_-(C-C-A-PCP)_2_-(C-A-PCP-E)_3_-(C-fkbH-PCP)_4_-(C-A-PCP-E-TE)_5_	Asn-Asn-Phe-GA-Tyr	Etzbach et al. (2014)
WAP-8294A	*Lysobacter*	*wapA-B*	12	(C-A-PCP)_1_-(C-A-PCP-E)_2_-(C-A-PCP)_3-4_-(C-A-PCP-MT-E)_5_-(C-A-PCP)_6_-(C-A-PCP-E)_7_-(C-A-PCP)_8_-(C-A-PCP-E)_9–11_-(C-A-PCP-MT)_12_-TE	Ser-Asn-Ser-Gly-(*N*me-Phe)-Leu-Orn-Glu-Asn-Trp-Orn-(*N*me-Val)	Zhang et al. (2011)
WBP-28479A1	*Lysobacter*	*wbpA-B*	11	(C-A-PCP)_1_-(C-A-PCP-E)_2_-(C-A-PCP)_3-4_-(C-A-PCP-MT-E)_5_-(C-A-PCP)_6_-(C-A-PCP-E)_7_-(C-A-PCP)_8_-(C-A-PCP-E)_9–10_-(C-A-PCP-TE)_11_	Val-Arg-Ser-Gly-(*N*me-Phe)-Leu-Arg-Glu-Val-Trp-Aba	Sang et al. (2019)
Lysocin	*Lysobacter*	*lesA-B*	12	(C-A-PCP)_1_-(C-A-PCP-E)_2_-(C-A-PCP)_3-4_-(C-A-PCP-MT-E)_5_-(C-A-PCP)_6_-(C-A-PCP-E)_7_-(C-A-PCP)_8_-(C-A-PCP-E)_9–10_-(C-A-PCP)_11_-(C-A-PCP-TE)_12_	Thr-Arg-Ser-Gly-Phe-Leu-Arg-Glu-Gln-Trp-Val/Ile-Thr	Panthee et al. (2016)

GA, glyceric acid; MT, methyltransferase.

During the genome mining conducted in the current review, 20 unknown BGCs with a hybrid PKS-NRPS system were identified that did not correspond to lipopeptides previously characterised in *Myxococcus* sp. For example, antiSMASH analysis showed a BGC present in *M. xanthus* and *M. hansupus*, that encodes for a lipopeptide with the structure FA-X-Gly-Thr-Asn-Phe-Orn-Orn-TE (Figure 4D; Supplementary Table S4). Moreover, antiSMASH analysis conducted in this review revealed that one species (*Myxococcus stipitatus*) may be a newly identified lipopeptide producer containing at least one BGC that putatively encodes for a lipopeptide (hybrid PKS-NRPS system).

### Cystobacter

Two *C. fuscus* strains were available on the NCBI database for genome mining analysis in the current study and seven BGCs encoding for a PKS-NRPS hybrid system were detected within this species. Additionally, the BGC involved in the biosynthesis of the cystomanamides was amongst these seven BGCs detected (Supplementary Table S4). The *ctm* BGC has been identified to encode for the production of the cystomanamides, where the *ctmA* gene in particular was found to encode for the complex protein that contain functional domains belonging to the fatty acid synthesis (Etzbach et al., 2014). The *ctmB*, *ctmC* and *ctmD* genes encode for NRPSs, with a predicted peptide sequence of Asn-Asn-Phe-GA-Tyr (where GA refers to glyceric acid) (Etzbach et al., 2014) (Table 2). The remaining six BGCs with a hybrid PKS-NRPS system did not correspond to lipopeptides previously characterised. For example, antiSMASH analysis conducted for this review showed a BGC present in *C. fuscus* DSM 52655 that putatively encodes for a lipopeptide with a NRPS gene cluster containing five modules. This unknown BGC putatively encodes for the sequence: FA-Gly-Gly-Glu-Pro-Ser-TE (Figure 4E).

### Lysobacter

A total of 36 genomes (10 different species) from the NCBI were analysed using antiSMASH and all species were found to contain at least one BGC encoding for a hybrid PKS-NRPS system (Supplementary Table S5). The BGCs for the WAP-8294A, WBP-28479A1, lysocins and tripropeptins compounds have been described in literature (Table 2). The BGC for the WAP-8294A compounds, is reported to contain a PKS region with three structural genes, where *wapC* encodes for the NRPS-associated protein MbtH, and *wap2* and *wap3* encode for two multi-module NRPSs. The latter two NRPSs are comprised of 12 modules [predicted peptide sequence of Ser-Asn-Ser-Gly-(*N*me-Phe)-X-X-X-Asn-X-X-(*N*me-Val)] in the WAP-8294A compounds (Table 2) (Zhang et al., 2011; Yue et al., 2022). The WAP-8294A BGC has been detected in *L. enzymogenes* (Zhang et al., 2011), while genome mining analysis in this review detected the WAP-8294A BGC in *L. capsica* (Supplementary Table S5)*.* Recently, Sang et al. (2019) identified and reported on the cryptic BGC for WBP-28479A1. The *wbp* BGC was identified to encode for two large NRPS genes with a total of 11 modules [predicted peptide structure of Val-Arg-Ser-Gly-(*N*me-Phe)-Leu-Arg-Glu-Val-Trp-Aba] (Table 2). The WBP-28479A1 BGC has been detected in *L. antibioticus* (Zhang et al., 2011), and was similarly detected in strains of this species using the genome mining analysis conducted for this review (Supplementary Table S5)*.*

For the lysocins, Panthee et al. (2016) sequenced the genome of *Lysobacter* sp. RH2180-5 and analysed the putative lysocin BGC. The study indicated that too large multi-modular NRPSs named *lesA* and *lesB*, containing 12 modules, were involved in the biosynthesis of lysocin E, where the predicted peptide sequence was Thr-Arg-Ser-Gly-Phe-Leu-Arg-Glu-Gln-Trp-Val/Ile-Thr (Table 2). The biosynthetic genes involved in tripropeptin biosynthesis have not been reported in *Lysobacter*; however, the tripropeptin BGC was reported in *Collimonas* (Yue et al., 2022). The tripropeptins BGC was identified in *Collimonas fungivorans* Ter331 and Ter6 genomes by Song et al. (2015) and contained three NRPS-related genes (*trpA*, *trpB,* and *trpC*) in the BGC comprised of 8 modules.

During the genome mining conducted in the current review, 16 unknown BGCs with a hybrid PKS-NRPS system were identified that did not correspond to lipopeptides previously characterised in *Lysobacter* species. For example, antiSMASH analysis indicated a BGC that putatively encodes for a novel lipopeptide with a PKS and four NRPSs gene clusters, comprising 11 modules in *Lysobacter gummosus* (Figure 4F). The predicted peptide structure of this lipopeptide is FA-Leu-Leu-X-Leu-Leu-X-Ile-Thr-Gly-Asp-Ser-TE (Figure 4F; Supplementary Table S5). Moreover, antiSMASH analysis also putatively identified seven species (such as *Lysobacter alkalisoli, Lysobacter soli* and *Lysobacter solisilvae* etc.) as new lipopeptide producers containing at least one BGC that putatively encodes for a lipopeptide (hybrid PKS-NRPS system).

## Discussion and conclusion

This review provided insight into the classification and chemical diversity of lipopeptides isolated from members of the underexplored *Serratia*, *Brevibacillus*, *Burkholderia*, *Myxococcus*, *Cystobacter,* and *Lysobacter* genera. Each family of lipopeptides generally contains numerous homologues and analogues, with differing antimicrobial properties. Apart from the unknown antimicrobial activities of the myxochromides, cystomanamides, malleipeptins/burkhomycin, “haereo” and “burrio” families, the majority of the lipopeptides from these bacterial genera exhibit potent activity and are thus valuable sources of clinically useful antibiotics or lead compounds. However, despite prominent antimicrobial activity exhibited by several lipopeptides from *Burkholderia* and *Serratia* species, limited information regarding the mode of action of these lipopeptides currently exists and further investigation is recommended. Moreover, apart from laterocidine and relacidines, the majority of the lipopeptides from *Brevibacillus* and *Lysobacter* have a general cell membrane disruption mechanism of action (in a menaquinone-dependent manner for lipopeptides from *Lysobacter*), which is considered advantageous as the development of resistance to lipopeptides is likely to be slow and limited (Cochrane and Vederas, 2016).

Based on the genome mining conducted within this review, it is apparent that many undiscovered BGCs with a hybrid PKS-NRPS system are present within the genomes of *Serratia*, *Brevibacillus*, *Burkholderia*, *Myxococcus*, *Cystobacter,* and *Lysobacter* species, suggesting that they are underexplored sources of novel lipopeptide families with potentially potent antimicrobial activity. Therefore, future lipopeptide research should focus on either prospecting for these bacterial species from their respective environmental habitats to isolate and characterise these novel lipopeptide families or utilise the genome mining data from this review for genetic engineering and heterologous expression to biosynthesise these novel lipopeptide families (circumventing the need for culture-based prospecting methods).
