# Supplementary material for: Underexplored bacteria as reservoirs of novel antimicrobial lipopeptides
Source: Front Chem. 2022 Oct 5;10:1025979. doi: 10.3389/fchem.2022.1025979 (PMC9581180; doi:10.3389/fchem.2022.1025979)
Supplement: Supplementary file 1 [file DataSheet1.docx]

**Supplementary Information**

**Underexplored bacteria as reservoirs of novel antimicrobial lipopeptides**

Tanya Clements-Decker^1^, Megan Kode^2^, Sehaam Khan^1^ and Wesaal Khan^2*^

^1^Faculty of Health Sciences, University of Johannesburg, PO Box 17011, Doornfontein, 2028, South Africa

^2^Department of Microbiology, Faculty of Science, Stellenbosch University, Private Bag X1, Stellenbosch, 7602, South Africa

**Table S1.** Summary of genome mining for potential lipopeptide biosynthetic gene clusters within *Serratia* species.

| **Lipopeptide** | **Peptide moiety based on BGC** | ***Serratia* spp. (no. strains)** | | | | | | | | | | | | | | | | |
| --- | --- | --- | --- | --- | --- | --- | --- | --- | --- | --- | --- | --- | --- | --- | --- | --- | --- | --- |
|  |  | *S. bockelmannii* (*n* = 1) | *S. ficaria* (*n* = 1) | *S. fonticola* (*n* = 8) | *S. grimesii* (*n* = 1) | *S. inhibens* (*n* = 1) | *S. liquefaciens* (*n* = 8) | *S. marcescens* (*n* = 105) | *S. nematodiphila* (*n* = 1) | *S. plymuthica* (*n* = 19) | *S. proteamaculans* (*n* = 2) | *S. quinivorans* (*n* = 2) | *S. rhizosphaerae* (*n* = 1) | *S. rubidaea* (*n* = 9) | *S. surfactantfaciens* (*n* = 1) | *S. symbiotica* (*n* = 2) | *S. ureilytica* (*n* = 9) | *Serratia* sp. (*n* = 24) |
| **Serrawettin W1** | FA-Ser-TE **OR** FA-X-Ser-TE | **X** |  | **X** | **X** | **X** | **X** | **X** | **X** | **X** | **X** | **X** | **X** | **X** | **X** | **X** | **X** | **X** |
| **Serrawettin W2** | FA-Leu-Ser-Thr-Phe-X-TE |  |  |  |  |  |  | **X** |  |  |  |  |  |  | **X** |  | **X** | **X** |
| **Stephensiolides** | FA-Thr-Ser-Ser-X-X-TE |  | **X** | **X** |  |  |  | **X** |  |  |  |  |  |  |  |  | **X** | **X** |
| **Unknown** | FA-Thr-Ser-X-X-TE |  |  |  |  |  |  | **X** |  |  |  |  |  |  |  |  | **X** |  |
| **Unknown** | FA-Thr-Ser-Leu-TE |  |  |  |  |  |  | **X** |  |  |  |  |  |  |  |  |  |  |
| **Unknown** | FA-Tyr-Ile-Leu-Val-Ser-TE | **X*** |  | **X** |  |  |  |  |  |  |  |  |  |  |  |  |  | **X** |
| **Unknown** | FA-Tyr-Leu-Val-Ser |  |  | **X** |  |  |  |  |  |  |  |  |  |  |  |  |  |  |
| **Unknown** | FA-Phe-Gly-TE |  |  |  |  |  | **X** | **X** | **X** |  | **X** | **X** | **X** | **X** |  |  | **X** | **X** |
| **Unknown** | FA-X-X-Cys-TE |  |  |  |  |  |  | **X** |  | **X** |  |  |  |  |  |  |  | **X** |
| **Unknown** | FA-X-X-Asp-TE |  |  |  |  |  |  | **X** |  |  |  |  |  |  |  |  |  |  |
| **Unknown** | FA-Val-X-X-Cys-TE |  |  |  |  |  |  | **X** | **X** |  |  |  |  |  |  |  | **X** | **X** |
| **Unknown** | FA-Ser-Phe-X-TE |  |  |  |  |  |  | **X** |  |  |  |  |  |  |  |  |  |  |
| **Unknown** | FA-Cys-Ala-Cys-Asn-Phe-Gly-TE |  |  |  |  |  |  | **X** |  |  |  |  |  |  |  |  | **X** | **X** |
| **Unknown** | FA-Ser-Leu-X-TE |  |  |  |  |  |  | **X** |  |  |  |  |  |  |  |  |  |  |
| **Unknown** | FA-Thr-Leu-X |  |  |  |  |  |  | **X** |  |  |  |  |  |  |  |  |  |  |
| **Unknown** | FA-Thr-Ser-X-Val-Ser |  |  |  |  |  |  |  |  |  |  |  |  |  |  |  |  | **X** |
| **Unknown** | FA-Leu-X |  |  |  |  |  |  | **X** |  |  |  |  |  |  |  |  |  |  |
| **Unknown** | FA-X-Cys |  |  |  |  |  |  |  | **X** |  |  |  |  |  |  |  |  |  |
| **Unknown** | FA-X-Phe-Asn-Asn-Thr-Val-Ohmal |  |  |  |  |  |  |  |  | **X** |  |  |  |  |  |  | **X** |  |
| **Unknown** | FA-Cys-Ala-Cys-Asp-TE |  |  |  |  |  |  |  |  | **X** |  |  |  |  |  |  |  |  |
| **Unknown** | FA-Ala-Leu-X-Ala |  |  |  |  |  |  |  |  |  |  |  |  |  |  |  |  | **X** |

FA – Fatty acid. No. – Number. *without PKSs.

**Table S2**. Summary of genome mining for potential lipopeptide biosynthetic gene clusters within *Brevibacillus* species.

| **Lipopeptide** | **Peptide moiety based on BGC** | ***Brevibacillus* spp. (no. strains)** | | | | | | |
| --- | --- | --- | --- | --- | --- | --- | --- | --- |
|  |  | *B. agri* (*n* = 1) | *B. brevis* (*n* = 7) | *B. composti*  (*n* = 2) | *B. formosus*  (*n* = 1 ) | *B. laterosporus*  (*n* = 6) | *B. parabrevis*  (*n* = 1) | *Brevibacillus* sp.  (*n* = 4) |
| **Tauramamide** | FA-Tyr-Ser-Leu-Trp-Arg **OR** FA-X-Ser-X-X-X-TE |  |  |  |  | **X** |  |  |
| **Bogorol** | FA-Thr-Phe-X-Ile-Ile-Ile-X-Ile-Ile-X-X-Leu-Ile-TE |  |  |  |  | **X** |  |  |
| **Brevicidine** | FA-Asn-Phe-Tyr-Orn-Orn-Gly-Orn-Tyr-Thr-Ile-Gly-Ser |  |  |  |  | **X** |  |  |
| **Relacidine** | FA-Ser-Tyr-Trp-Orn-Orn-Gly-Orn-Trp-Thr-Ile-Gly-Ser-Gly |  |  |  |  | **X** |  |  |
| **Laterocidine** | FA-Ser-Phe-Tyr-Orn-Orn-Gly-Orn-Tyr-Thr-Ile-Asn-Gly-Gly |  |  |  |  | **X** |  |  |
| **Tyrocidine + FA** | FA-Phe-Pro-X-Phe-Asn-Gln-Tyr-Val-Orn-Leu or FA-Phe-Pro-Phe-Leu-Asn-Gln-Tyr-Val-Orn-Leu-TE |  | **X** |  |  |  |  | **X** |
| **Unknown** | FA-Phe-Pro-Phe-Phe-Asn-Asp-X-Val-Orn-Leu-X-Ser-X-X-X-X-Ohmal-X |  |  |  |  | **X** |  |  |
| **Unknown** | FA-Asn-Gly-Cys-Ohmal-Ohmal-Ohmal-Gly-X-Ccmal-Ser-Phe-Pro-Phe-Phe-Asn-Gln-Tyr-Val-Orn-Leu | **X** |  |  |  |  |  |  |
| **Unknown** | FA-Asn-Gly-Mal-Ser-Ser-Ohmal-Thr-Gly-Cys-Ohmal-Ohmal-Ohmal-Gly-X-Ohmal-Gly-Ccmal-Ccmal-Ccmal-X-Ccmal-Ccmal-Ser-X-X-Phe-Pro-Phe-Phe-Asn-Gln-Tyr-Val-Orn-Leu |  |  |  | **X** |  | **X** | **X** |
| **Unknown** | FA-Ile-Ser-Cys-Cys-Cys-Cys-Asn-Gly-Mal-Ser-Ser-Ohmal-Thr-Gly-Cys-Ohmal-Ohmal-X-PKS-X-Ohmal-Gly-Ccmal-Ccmal-Ccmal-Gly-Ccmal-Ser-X-X-X |  | **X** |  |  |  |  |  |
| **Unknown** | FA-Pro-Leu-Pro-Leu-Ile-Leu-X-Leu-Ile-Thr-X-Leu-Asp-Ile-Tyr-Leu-Ccmal-X-Pro-Phe-Phe-Asn-Asp-X-Val-Orn-Leu-X-Ohmal-X |  |  |  |  | **X** |  |  |
| **Unknown** | FA-Pro-Leu-Pro-Leu-Ile-Leu-X-Leu-Ile-Thr |  |  |  |  | **X** |  |  |
| **Unknown** | FA-Orn-X-Phe |  | **X** |  |  |  |  |  |
| **Unknown** | FA-Cys-Ser-Cys-Ile-(*N*me-Cys)-TE |  |  | **X** |  |  |  |  |
| **Unknown** | FA-Glu-Ser-Val-Val-X-Phe-Orn **OR** FA-Glu-Ser-Ile-X-Phe-Orn |  | **X** |  |  |  |  |  |

**Table S3**. Summary of genome mining for potential lipopeptide biosynthetic gene clusters within *Burkholderia* species.

| **Lipopeptide** | **Peptide moiety based on BGC** | ***Burkholderia* spp. (no. strains)** | | | | | | | | | | | | | | | | | | | | |
| --- | --- | --- | --- | --- | --- | --- | --- | --- | --- | --- | --- | --- | --- | --- | --- | --- | --- | --- | --- | --- | --- | --- |
|  |  | *B. ambifaria* (*n* = 8) | *B. cenocepacia* (*n* = 12) | *B. cepacia* (*n* = 12) | *B. contaminans* (*n* = 6) | *B. gladioli* (*n* = 9) | *B. glumae* (*n* = 12) | *B. lata* (*n* = 3) | *B. mallei* (*n* = 8) | *B. mayonis* (*n* = 2) | *B. oklahomensis* (*n* = 5) | *B.* *perseverans* (*n* = 1) | *B. plantarii* (*n* = 2) | *B. pseudomallei* (*n* = 25) | *B. pyrrocinia* (*n* = 4) | *B. savannae* (*n* = 2) | *B. seminalis* (*n* = 2) | *B. stabilis* (*n* = 3) | *B. stagnalis* (*n* = 1) | *B. thailandensis* (*n* = 7) | *B. ubonensis* (*n* = 8) | *B. vietnamiensis* (*n* = 6) |
| **Icosalide** | FA-Leu-Ser-FA-Ser-Leu |  |  |  |  | **X** |  |  |  |  |  |  |  |  |  |  |  |  | **X** |  |  |  |
| **Malleipeptin** | FA-Ser-X-Ser-X-Thr-Leu-X-Thr-Thr-X-Gly-Val-TE |  |  |  |  |  |  |  |  |  |  |  |  | **X** |  |  |  |  |  |  |  |  |
| **Haereogladin** | FA-Thr-Thr-X-X-X **OR** FA-Thr-Thr-Leu-Ile-X **OR** FA-Thr-Thr-X-X-Pro |  |  |  |  | **X** |  |  |  |  |  | **X** | **X** |  |  |  |  |  |  |  |  |  |
| **Burriogladin** | FA-Thr-Pro-Glu-Ala-X-X-Pro-TE |  |  |  |  | **X** |  |  |  |  |  |  |  |  |  |  |  |  |  |  |  |  |
| **Burrioglumin** | FA-Dhb/Thr-Pro-Ser-Ala-Val/Leu-Phe-Pro-Thr OR  FA-Thr-Pro-Ser-Ala-X-X-Pro-TE |  |  |  |  |  | **X** |  |  |  |  |  | **X** |  |  |  |  |  |  |  |  |  |
| **Unknown** | FA-Thr-Thr-X-Thr-Gln-Gly-Thr-X-*N*me-Thr-X-TE |  |  |  |  |  |  |  |  | **X** | **X** |  |  |  |  |  |  |  | **X** |  | **X** |  |
| **Unknown** | FA-Leu-Leu-X-X-X-TE |  |  |  |  | **X** |  |  |  | **X** | **X** |  |  | **X** |  |  |  |  |  |  |  |  |
| **Unknown** | FA-Ser-X-Gly-Val-TE |  |  |  |  |  |  |  | **X** |  |  |  |  | **X** |  |  |  |  |  |  |  |  |
| **Unknown** | FA-Asn-Ser-X-Phe-X-Mal-Ser-Leu-Ser-Ser-Leu-TE |  |  | **X** |  |  |  |  |  |  |  |  |  |  |  |  |  |  |  |  |  |  |
| **Unknown** | FA-Cys-Cys-TE | **X** | **X** | **X** | **X** |  |  | **X** |  |  |  | **X** | **X** |  |  |  | **X** | **X** |  |  |  |  |
| **Unknown** | FA-Cys-Ala-Cys-X-TE |  |  |  |  |  |  |  |  |  |  |  |  |  |  | **X** |  |  |  | **X** |  |  |
| **Unknown** | FA-Gly-Ser-Gly-Asn-Gln-Val-Mal-X-Asn-Ile-Thr-Phe-Thr-*N*me-X-Gly-Ser-Asn-X-*N*me-Ala-*N*me-Phe-Ohmal-Pro-Gly-Gly-TE |  |  |  |  | **X** |  |  |  |  |  |  |  |  |  |  |  |  |  |  |  |  |
| **Unknown** | FA-X-X-Gly-X-X-Ser-Ser-X-TE |  |  |  |  | **X** | **X** |  |  |  |  | **X** |  |  |  |  |  |  |  |  |  |  |
| **Unknown** | FA-Ser-Dab-X-Dab-Dab-Tyr-Thr-Asp-Thr-TE |  |  |  |  | **X** |  |  |  |  |  |  |  |  |  |  |  |  |  |  |  |  |
| **Unknown** | FA-Ser-X-X-Phe-Asn-Ser-TE | **X** |  | **X** | **X** |  |  | **X** |  |  |  |  |  |  | **X** |  |  |  |  |  | **X** | **X** |
| **Unknown** | FA-Thr-Orn-X-Ccmal-Tyr-TE |  |  |  |  |  |  |  |  |  |  | **X** | **X** | **X** |  |  |  |  |  |  |  |  |
| **Unknown** | FA-Asp-Gln-Cys-Gly-Ohmal-Ala-TE |  |  |  |  |  |  |  |  |  |  |  |  |  |  |  |  |  |  | **X** |  |  |
| **Unknown** | FA-Asn-Gly-X-Ala-X-X-Ala-Cys-TE |  |  |  |  |  |  |  |  |  |  |  |  |  |  |  |  |  |  | **X** |  |  |
| **Unknown** | FA-X-X-Pro |  |  |  |  |  |  |  |  |  |  |  |  |  |  |  |  |  |  |  | **X** |  |
| **Unknown** | FA-Pro-Ser |  |  |  |  |  |  |  |  |  |  | **X** |  |  |  |  |  |  |  |  |  |  |
| **Unknown** | FA-X-Orn |  |  |  |  |  |  |  |  |  |  | **X** |  |  |  |  |  |  |  |  |  |  |
| **Unknown** | FA-Thr-TE |  |  |  |  |  |  |  | **X** |  |  |  |  |  |  |  |  |  |  |  |  |  |
| **Unknown** | FA-X-Asp-Ser-X |  |  |  |  |  |  |  | **X** |  |  |  |  |  |  |  |  |  |  |  |  |  |
| **Unknown** | FA-Ser-Val-X |  |  |  |  | **X** |  |  |  |  |  |  |  |  |  |  |  |  |  |  |  |  |

**Table S4**. Summary of genome mining for potential lipopeptide biosynthetic gene clusters within *Myxococcus* and *Cystobacter* species.

| **Lipopeptide** | **Peptide moiety of BGC** | ***Myxococcus* spp. (no. strains)** | | | | | ***Cystobacter* spp. (no. strains)** |
| --- | --- | --- | --- | --- | --- | --- | --- |
|  |  | *M. fulvus*  (*n* = 1) | *M. hansupus*  (*n* = 1) | *Myxococcus* sp. (*n* = 1) | *M. stipitatus*  (*n* = 1) | *M. xanthus*  (*n* = 12) | *C. fuscus*  (*n* = 2) |
| **Myxochromide A** | FA-Thr-Pro-Leu-Pro-Phe-Gln |  |  |  |  | **X** |  |
| **Myxochromide C** | FA-Thr-Pro-Leu-Pro-X |  | **X** |  |  | **X** |  |
| **Cystomanamide** | FA-Asn-Asn-Phe-GA-Tyr |  |  |  |  |  | **X** |
| **Unknown** | FA-X-Ohmal-Ohmal-Ser-Val-Val-Val-Val-Val-Pro | **X** |  |  |  |  |  |
| **Unknown** | FA-Tyr-Mal-X-Gln-Gln-Val-Asp-Val | **X** |  |  |  |  |  |
| **Unknown** | FA-Pro-Ohmal-Asn-X-Val-X | **X** |  |  |  |  |  |
| **Unknown** | FA-Val-Pro-Pro | **X** |  |  |  |  |  |
| **Unknown** | FA-X-Mal-X-Val-X-Ser |  | **X** |  |  |  |  |
| **Unknown** | FA-Ser-X-X-Val-Ser-X-Mal-Ser-Val-X-X **OR** FA-Ser-X-Val-Ser-X-Mal-Ser-X-X-X-X |  | **X** |  |  | **X** |  |
| **Unknown** | FA-X-Ser-Ile-Val-Mal-Phe-X-Val-Val-Val-Asp-Val-Val-Val-Ala-X |  | **X** |  |  |  |  |
| **Unknown** | FA-Thr-X-Val-Ser-X-Mal-Ser-Val-X-Asp |  |  | **X** | **X** |  |  |
| **Unknown** | FA-X-Mal-X-Asn-Pro-Val-Asp-Val |  |  | **X** | **X** |  |  |
| **Unknown** | FA-X-Ohmal-Ser-Gly-Val-Val-Val-Val-Ser-Pro-Tyr-X-Tyr-Lys-Pro |  |  | **X** | **X** |  |  |
| **Unknown** | FA-Orn-Thr-Asn-Tyr-Orn-Gly-Mal-X-Ser-Gly-Gly-Phe-Gln-Gln-Val-X-Val-Gln-Glu-Gln-X |  |  | **X** |  |  |  |
| **Unknown** | FA-Asn-Val-Glu-Gln-Ala-Ala-Ccmal-X-Orn-Thr-Asn-Tyr-Orn-Gly-Mal-X-Ser-Gly-Gly-Phe-Gln-Gln-Val-X-Val-Gln-Glu-Gln-X |  |  |  | **X** |  |  |
| **Unknown** | FA-Val-X-X-Phe-X |  |  | **X** | **X** |  |  |
| **Unknown** | FA-X-X-X-X-Asn-X |  |  |  |  | **X** |  |
| **Unknown** | FA-X-Mal-X-Asp-Val-Asp-Val |  |  |  |  | **X** |  |
| **Unknown** | FA-X-Gln-Leu-Leu-X |  |  |  |  | **X** |  |
| **Unknown** | FA-X-Gly-Thr-Asn-Phe-Orn-Orn |  | **X** |  |  | **X** |  |
| **Unknown** | FA-X-Gly-Thr-Asn-Phe-Orn-Orn-Ser-Val-Gly-Mal-X |  |  |  |  | **X** |  |
| **Unknown** | FA-Gln-X |  |  |  |  | **X** |  |
| **Unknown** | FA-Thr-Val-Val-X-X-Gly-Mal |  |  |  |  | **X** |  |
| **Unknown** | FA-Gly |  |  |  |  |  | **X** |
| **Unknown** | FA-Thr-Leu |  |  |  |  |  | **X** |
| **Unknown** | FA-Gly-Gly-Glu-Pro-Ser |  |  |  |  |  | **X** |
| **Unknown** | FA-Tyr-Mal-Pro-Pro-Gln-Asp |  |  |  |  |  | **X** |
| **Unknown** | FA-X-Asn-X-Val-X-Ile-Ser-Ohmal |  |  |  |  |  | **X** |
| **Unknown** | FA-Tyr-Mal-X-Phe-X-Glu |  |  |  |  |  | **X** |

| **Lipopeptide** | **Peptide moiety of BGC** | ***Lysobacter* spp. (no. strains)** | | | | | | | | | | |
| --- | --- | --- | --- | --- | --- | --- | --- | --- | --- | --- | --- | --- |
|  |  | *L.* *alkalisoli*  (*n* = 1) | *L. antibioticus*  (*n* = 2) | *L. arenosi*  (*n* = 1) | *L. capsica*  (*n* = 4) | *L. ciconiae*  (*n* = 1) | *L. enzymogenes* (*n* = 4) | *L. gummosus*  (*n* = 3) | *L. maris* (*n* = 1) | *L. soli* (*n* = 1) | *L.* *solisilvae*  (*n* = 2) | *Lysobacter sp.*  (*n* = 5) |
| **WAP-8294A2 / Lotilibcin** | FA-Ser-Asn-Ser-Gly-(Nme-Phe)-Leu-Orn-Glu-Asn-Trp-Orn-(Nme-Val) |  |  |  | **X** |  |  |  |  |  |  |  |
| **WBP-28479A1** | FA-Val-Arg-Ser-Gly-(*N*me-Phe)-Leu-Arg-Glu-Val-Trp-Aba |  | **X** |  |  |  |  |  |  |  |  |  |
| **Unknown** | FA-X-Mal-Gly | **X** |  | **X** |  |  |  |  |  | **X** | **X** | **X** |
| **Unknown** | FA-X-Val-Leu-Thr |  | **X** |  |  |  |  |  |  |  |  |  |
| **Unknown** | FA-X-Val-X-X |  | **X** |  |  |  |  |  |  |  |  |  |
| **Unknown** | FA-Orn |  |  |  | **X** |  | **X** | **X** |  |  |  | **X** |
| **Unknown** | FA-X-Ala-X-Thr-Cys-Ala-Ohmal-Thr-Cys-X-Ser-Ser-Cys-Ser-X-Ser-Asn-Ser-Gly-Phe-X-X-X-Asn-X-X-Val |  |  |  | **X** |  |  |  |  |  |  |  |
| **Unknown** | FA-X-Gly-X-Gly |  |  |  | **X** |  |  |  |  |  |  |  |
| **Unknown** | FA-Val-Mal-Asn-Gly |  |  |  |  | **X** |  |  |  |  |  |  |
| **Unknown** | FA-Gly-Ohmal-Mal |  |  |  |  |  | **X** |  |  |  |  |  |
| **Unknown** | FA-Ccmal-Ser-X-Ser-X |  |  |  |  |  | **X** |  |  |  |  |  |
| **Unknown** | FA-X-Tyr-X-X-Cys-Gly-Gly-Asn-Asn-Asn-Ohmal-Asn-Asn-Asp-Thr |  |  |  |  |  | **X** |  |  |  |  |  |
| **Unknown** | FA-Leu-Leu-X-Leu-Leu-X-Ile-Thr-Gly-Asn-Ser |  |  |  |  |  |  | **X** |  |  |  |  |
| **Unknown** | FA-Val-Mal-Val |  |  |  |  |  |  |  | **X** |  |  |  |
| **Unknown** | FA-X-Asp-Asp-Val-Asp-Tyr-Asn-Glu-Tyr-Ala-X |  |  |  |  |  |  |  |  |  | **X** |  |
| **Unknown** | FA-X-Thr-X-X-Ala |  |  |  |  |  |  |  |  |  |  | **X** |
| **Unknown** | FA-X-Gly |  |  |  |  |  |  |  |  |  |  | **X** |
| **Unknown** | FA-Val-Mal-Gly-Gly |  |  |  |  |  |  |  |  |  |  | **X** |

**Table S5**. Summary of genome mining for potential lipopeptide biosynthetic gene clusters within *Lysobacter* species.
